# Mucosal IL-36 is a defining feature of severe paediatric bronchiolitis

**DOI:** 10.1016/j.mucimm.2026.01.012

**Published:** 2026-04

**Authors:** Megan V.C. Barnes, Sophie W.H. Stretch, Dawid Swieboda, Claudia Efstathiou, Fiona J. Culley, Trevor T. Hansel, Sam Strickson, Xavier Romero-Ros, E. Suzanne Cohen, Simon Nadel, Peter J.M. Openshaw, Ryan S. Thwaites

**Affiliations:** a National Heart and Lung Institute, Imperial College London, London, UK; b Bioscience Asthma and Skin Immunity, Research and Early Development, Respiratory & Immunology, BioPharmaceuticals R&D, AstraZeneca, Cambridge, UK; c Pediatric Intensive Care Unit, St. Mary’s Hospital, Imperial College Healthcare Trust, London, UK

**Keywords:** Respiratory syncytial virus, Bronchiolitis, Inflammation, Mucosa

## Abstract

**Rationale:**

Bronchiolitis is the commonest cause of hospital admission in children under the age of 1 year, most cases being due to respiratory syncytial virus (RSV) infection. The mechanisms causing infantile bronchiolitis are incompletely understood but include a deficient mucosal interferon response, neutrophilic inflammation and enhanced mucosal Type-2 responses.

**Objectives:**

We sought to determine the mucosal immune processes associated with severe paediatric bronchiolitis.

**Methods:**

We performed transcriptomic analyses on mucosal samples from infants hospitalized with Moderate (*n* = 48) and Severe (*n* = 40) bronchiolitis. Differential expression and regression analyses determined genes associated with different severity categories. Responses were modelled *in vitro* using air–liquid interface human nasal epithelial cell culture models.

**Measurements and main results:**

We confirmed weakened interferon-associated signalling in severe RSV and non-RSV bronchiolitis but unexpectedly found elevated IL-36α (an IL-1 family cytokine implicated in chronic inflammatory diseases) early in infection. Conversely, *IL36A* was decreased in whole blood during severe RSV, suggesting that this association is unique to the mucosa. In human nasal epithelial cells grown *in vitro* under air–liquid interface we found IL-36α to be produced by epithelial cells during RSV infection and that its secretion is enhanced by neutrophils.

**Conclusions:**

These findings implicate mucosal IL-36α as a dominant feature of severe paediatric bronchiolitis.

**One sentence summary:**

Mucosal transcriptomics identifies IL-36 secretion as a feature of life-threatening paediatric RSV and all-cause bronchiolitis associated with interferon response failure.

## Introduction

Respiratory syncytial virus (RSV) is the most common cause of acute lower respiratory tract infections (ALRI) in infants and children up to the age of two,^1^ and those under the age of six months are at greatest risk of hospitalisation from RSV infection.^2^ RSV also threatens the elderly and immunocompromised, whilst presenting as common-cold symptoms in healthy adults.^3^ The therapeutic and vaccine landscape for paediatric RSV is rapidly changing; the anti-RSV F-protein monoclonal antibodies Palivizumab^4^ or Nirsevimab,^5^ or maternal vaccination^6^, ^7^ are available to prevent severe disease. A recently licensed pre-F maternal vaccine, Abrysvo, enhances protection from severe RSV infection in the first few months of life.^8^ Despite these advances in prophylaxis, there are no specific treatments for bronchiolitis and the mucosal mechanisms underpinning the immunopathogenic response to RSV and other respiratory viruses are incompletely understood. Most remarkably, viral load does not correlate with disease severity and may even show an inverse relationship with disease, whereby those with severe infection had a lower viral load than those with milder disease.^9^, ^10^ These findings indicate the importance of the inflammatory response in causing disease during viral bronchiolitis.

The epithelium is the primary site to initiate innate immune responses following RSV infection, which occurs upon activation of pattern recognition receptors (PRRs) including Toll-like receptors (TLRs), NOD-like receptors (NLRs), or RIG(I)-like receptors (RLRs).^11^, ^12^ This results in the production of pro-inflammatory mediators, including interferons (IFNs) and interferon-stimulated genes (ISGs).^13^ Activation of epithelial PRRs results in the chemoattraction and activation of immune cells that are important for combatting the infection at the respiratory mucosa.^14^ High levels of neutrophil infiltration have been associated with RSV bronchiolitis^15^, ^16^; although neutrophils mediate antimicrobial effects on RSV-infected cells, heightened neutrophilic responses contribute to epithelial damage and lung morbidity.^17^ This has been modelled in airway epithelial cells cultured under air–liquid interface (ALI) conditions, where neutrophils were shown to damage the epithelium during RSV infection.^18^

There are immunological markers of severity that have provided some mechanistic insight into disease progression. We previously reported that severe RSV infection is characterised by a reduced mucosal interferon response, but heightened IL-17 and mucin expression.^9^ Mucin expression is indicative of an increase in mucus production, a hallmark physiological feature of bronchiolitis.^19^, ^20^ There is also evidence for enhanced CD14+/CD33 + myeloid cells in severe bronchiolitis.^21^ Using a combination of transcriptomics on upper-airway samples and 3D epithelial cell cultures, we aimed to identify additional inflammatory markers of severe RSV infection and unexpectedly identified IL-36 as a previously undescribed key factor.

## Materials and methods

### Patient recruitment and sampling

Ethical approval for sample collection was granted by the Research Ethics Committee (REC) and all patients, or parents/guardians of patients, provided informed written consent. Infants and children up to the age of two years, hospitalised for bronchiolitis at St Mary’s Hospital, were recruited to the RSVSAM (REC number 15/WM/0343) and ELLI (REC number 18/LO/1570) studies between 2016 and 2018, and 2018 and 2020, respectively. Healthy control children from RSVSAM were recruited from ophthalmology or fracture clinics and had no symptoms of respiratory infection. Healthy control infants were recruited if they were admitted to hospital for day surgery and presented with no sign of respiratory infection.

Patients within the RSVSAM study were defined as having either moderate or severe bronchiolitis, depending on their peak hospitalisation (moderate: accident and emergency (A&E)/pediatric wards, severe: Pediatric intensive care unit (PICU) admission). Respiratory samples were collected from all participants, including nasosorption (NSAM), nasopharyngeal aspirate (NPA) and bronchial aspirate (BAsp) as previously described.^9^ Aliquots of each sample were either kept neat or mixed with an equal volume of RNA Shield^TM^ (Zymo Research) for mediator analysis or RNA extraction, respectively.

### Analysis of patient samples

Diagnosis of respiratory infection and co-infection was confirmed by multiplex PCR assay, as previously described.^9^ Total RNA was extracted from banked NPAs and NSAMs according to manufacturer’s instructions (Qiagen; RNeasy Plus Micro Kit (50); #74034) and was analysed for both concentration and quality (260/230 and 260/280 ratios) using Nanodrop and Agilent 2100 Bioanalyser (Agilent Technologies; Agilent RNA 6000 Nano Kit (#5067-1511). NPA samples with > 10 ng/µL RNA, and of sufficient quality (DV200 score > 50%, as measured by Bioanalyser), were selected for downstream transcriptomic analyses. Transcriptomic profiling was performed on 80 ng RNA using NanoString host response panel (NanoString; nCounter Human Host Response Panel; #XT-HHR-12), to study 785 transcripts covering innate and adaptive immune responses, normalised to 12 internal reference genes. Any samples that passed the NanoString QC, and had > 30% probes bound, were included in the final analysis (Fig. E1). Total protein of NPAs and NSAMs was measured (ThermoFisher; Pierce^TM^ BCA Protein Assay Kit; #23225), according to manufacturer’s instructions. Protein levels of IL-36α (R&D Systems; Human IL-36 alpha/IL-1F6 DuoSet ELISA; #DY1078-05), IL-36β (R&D Systems; Human IL-36 beta/IL-1F8 DuoSet ELISA; #DY1099-05) and IL-36γ (Adipogen Life Sciences; #AG-46B-0010) were measured in neat NPAs, NSAMs and BAsps.

### RSV viral stock production

Cultured HEp-2 cells (ATCC) were infected with RSV-A2 in serum-free media at an MOI (multiplicity of infection) of 0.1 and incubated for two hours. The media was then switched to DMEM supplemented with 10% FCS and left for 24 h at 37°C. Media was then changed to DMEM + 2% FCS to allow the infected cultures to be maintained until five days post-infection (DPI), when cytopathic effects could be observed. The infected cells were sonicated and centrifuged to collect the supernatant containing the RSV stocks, which were snap frozen in liquid nitrogen. Pooled viral stocks were quantified using a focus forming unit (FFU) quantification assay.^22^

### HNEC isolation, culture and infections

To obtain human nasal epithelial cells (HNECs), brushings were performed on healthy adults by rotating a HydraFlock Standard Tip Nasal Swab (Medical Wire; #MW818) one-to-two inches inside the nasopharynx, as previously described.^23^, ^24^ The brushes were immediately placed in PneumaCult^TM^-Ex Plus Basal Medium (StemCell; #05040) supplemented with 1% Penicillin/Streptomycin and 50X Ex Plus Medium Supplement (StemCell; #05042). Cells were then dislodged from the brush by agitation and seeded onto a T25 flask coated with Type I Bovine Collagen Solution (Sigma; #804622; 1:60). Cells were passaged once, onto a T75 flask, before being moved into Transwells for air–liquid interface (ALI) culture. To prepare the basal cells for ALI culture, they were plated on 6.5 mm Transwells (Polyester membranes with 0.4 µm pores; StemCell; #38024) at a seeding density of 1 × 10^5^ and were maintained as submerged cultures until confluency was reached (typically 2–7 days), both apical and basolateral media were changed every two days. To initiate ALI conditions, the apical and basolateral media were removed, and PneumaCult^TM^-ALI Basal Medium was added to the basolateral compartment, leaving the apical side of the culture exposed to air. After two weeks at ALI, mucus was washed off the cultures weekly using PBS. Cultures were deemed ready for experiments when extensive cilia coverage was apparent, and transepithelial electrical resistance (TEER; a measure of tight junction integrity) was > 500Ω.^25^

### Inverted HNEC co-culture with neutrophils

HNECs from healthy adults for use in neutrophil co-cultures were purchased from Epithelix. Cells were thawed in a 37°C water bath, spun down to concentrate to 2 × 10^6^/mL and 50 μL (1 × 10^5^ final seeding density) seeded onto an inverted collagen coated 6.5 mm Transwell. These were left to adhere for four hours before the plate was turned upright, and both Transwell and basolateral compartment supplied with PneumaCult^TM^-Ex Plus Basal Medium (StemCell; #05040). The media was replaced the following day to remove any cells not adhered to the bottom of the Transwell, and then cells were maintained until confluency. The cells were airlifted by removing the media from the basolateral compartment and PneumaCult^TM^-ALI Basal Medium was added to the Transwell. After two weeks the cultures were washed with warm PBS. Cultures were maintained for four weeks at ALI, then TEER readings were made and cultures used for experiments, with a final epithelial cell count averaging 2 × 10^6^ by full differentiation. For neutrophil isolation, whole blood was collected from healthy adults (commercially sourced from Cambridge BioScience, ethical approvals provided by Research Donors ltd.) and neutrophils isolated no more than four hours post-venepuncture using the negative-selection EasySep^TM^ Direct Human Neutrophil Isolation Kit (StemCell; #100-0404) to isolate untouched neutrophils. Following isolation, neutrophils were immediately counted, viability confirmed to be > 95%, and 500 μL added to the inverted HNEC cultures at a density of 4 × 10^5^/mL, at a neutrophil:epithelial cell ratio of 1:5.

### RSV infection of upright 3D HNEC cultures

The total HNEC number per Transwell was determined by dissociating cells from one well and counting using a Countess cytometer (Countess 3 Automated Cell Counter; ThermoFisher). Prior to infection, stocks of RSV A2 were diluted in serum-free DMEM at an MOI 0.1. A brief wash of the apical surface was performed with sterile PBS, and the basolateral media was replaced. Fully differentiated HNECs, maintained under ALI, were infected with RSV at an MOI of 0.1 for two hours and mock infections with serum-free DMEM were used as controls. Following infection, the apical surface was washed twice with sterile PBS. Apical washes were performed daily from 0 to 5DPI by adding PBS to the apical surface and incubating for 45 min before collection. The basolateral medium was collected daily 0-5DPI; the cell lysates were collected 1-5DPI using Buffer RLT+ (Qiagen; #79216) supplemented with β-Mercaptoethanol (β-Me). All samples collected were stored at −80°C until analysis.

### RSV infection of inverted 3D HNEC cultures

To model the influence of neutrophils on epithelial RSV infection, an inverted HNEC culture model was employed where HNECs were cultured on the underside of a transwell insert with 0.4 µm pores.^26^ Cells were counted and as with upright cultures were infected with a final MOI of 0.1. The cells were washed immediately prior to infection by adding warm PBS to the basolateral compartment. Infection lasted two hours, and then the cells were washed again post-infection. Neutrophils (2 × 10^5^) were added to the RSV-infected cultures either immediately post-infection, or 20 h post-infection. Cultures ended after a total infection duration of 24 h. The 0.4 µm pore size was chosen to prevent neutrophil migration into the apical epithelial layer. Apical washes, basolateral supernatant and HNEC cell lysates were collected and stored at −80°C until analysis.

### Stimulation of HNEC cultures by cytokines/LPS

For basolateral stimulation of the HNECs maintained under ALI conditions, recombinant (r)IL-17A (R&D Systems; #317-ILB-050) was diluted to 50 ng/mL (1:2000) in 500 µL serum-free DMEM. Ultrapure LPS (InvivoGen; #tlrl-peklps) was diluted to 100 ng/mL in 300 µL serum-free DMEM for stimulation via the apical surface. HNECs were stimulated with either rIL-17A or LPS, or both, with or without additional RSV (MOI 0.1). All stimulations lasted two hours, with the basolateral medium changed and apical washes collected at 0-2DPI, and the cell lysate collected at 2DPI.

### Stimulation of HNEC cultures by RSV NPAs

To stimulate the HNEC cultures with NPAs, we selected six cell-free supernatants of NPAs from infants with severe bronchiolitis, to focus on the effect of soluble mediators. Total protein concentrations in the NPAs were normalised to 10 ng/mL in serum-free DMEM, and 500uL was added to the basolateral compartment of the Transwell. All stimulations were left for 2 h. After which, the basolateral medium was replaced, and apical side washed with PBS. The basolateral medium and apical washes were collected 0-4DPI, and the cell lysate was collected at 4DPI.

### Analysis of RSV-infected HNEC cultures

Viral RNA was isolated from apical washes (MagMAX^TM^-96 Viral RNA Isolation Kit; Applied Biosystems; #AM1836) and cell lysates and RSV-A nucleocapsid gene copies quantified using by RT-qPCR (Primer Design Ltd; #Path-RSV-A-standard). Total eukaryotic RNA was isolated from the cell lysate (Qiagen; RNeasy Plus Micro Kit (50); #74034) and was converted to cDNA (SuperScript VILO cDNA Synthesis Kit; Invitrogen; #11754050). Expression of characteristic genes of mucus-producing cells, basal cells and ciliated epithelial cells were determined by TaqMan real-time PCR on a 7500 Real Time PCR System using the following assays: *MUC5AC* (Hs01365616_m1), *MUC5B* (Hs00861595_m1), *FOXJ1* (Hs00230964_m1), *P63* (Hs00922561_m1), *KRT5* (Hs00361185_m1), *PIGR* (Hs00922561_m1). The expression of IL-36 isoforms was also measured by TaqMan qPCR assays: *IL36A* (Hs00205367_m1) and *IL36G* (Hs00219742_m1). Gene expression was normalised to *GAPDH* (Hs02786624_g1). Total RNA was analysed using the NanoString host response panel (nCounter Human Host Response Panel; NanoString; #XT-HHR-12), to identify the key markers of an epithelial response to RSV infection. Protein levels of IL-36α/IL-1F6 (R&D Systems; #DY1078-05), IL-36γ/IL-1F9 (Adipogen Life Sciences; #AG-46B-0010), and CXCL10/IP-10 (R&D Systems; #DY266) were measured using commercial ELISA kits. The detection of a further ten protein mediators (IFN-γ, IL-1β, IL-2, IL-4, IL-6, IL-8, IL-10, IL-12p70, IL-13 and TNF-α) were measured in NPAs using the Meso Scale Discovery (MSD) V-PLEX pro-inflammatory panel 1 human kit (MSD, #K15049D-2). Antibody staining for confocal microscopy was performed on mock- and RSV-infected cultures at 1DPI. Cultures were washed and incubated in warm 4% paraformaldehyde, followed by 0.2% Triton X-100. The primary antibodies were diluted in 0.5% BSA at a concentration suggested by the manufacturer: MUC5B (Merck, #292M-97) 1:250; RSV F protein (ThermoFisher, #MA1-7286) 1:10). The secondary antibodies were also diluted in 0.5% BSA: Anti-Rabbit – CF568 (Merck, #SAB4600084-50UL) 1:2000; Anti-Mouse – AF647 (Biolegend, #405322) 1:500. For mounting media, the ProLong^TM^ gold antifade DAPI stain was used, and coverslips were stored in the dark at 4°C until analysis. Cultures were visualised on a Leica SP8 Inverted Microscope.

### Data analysis

Data derived from NanoString, including differential gene expression analysis, gene set analysis (GSA), and cell type profiling, was performed using nSolver advanced analysis software. Raw gene counts were normalised to a subset of housekeeping genes under the nSolver advanced analysis. False discovery rate (FDR) correction was performed on the differential gene expression analysis using the Benjamini-Yekutieli FDR procedure. Data was then exported, and figures produced in RStudio (v2023.12.0 + 369), using packages: *ggplot2* (v3.4.4), *tidyverse* (v2.0.0), *EnhancedVolcano* (v1.12.0), *ggpubr* (v0.6.0) and *dplyr* (v1.1.4). Factor analysis of mixed data (FAMD) was also performed in RStudio (v2023.12.0 + 369), using packages *factoextra* (v1.0.7) and *FactoMineR* (v2.9). Included in the FAMD dataset was the normalised expression of the top 20 DEGs (for ease of visualisation) from the differential gene expression analysis, as well as clinical demographics. For analysis of statistical significance of cell markers, or protein analysis, between moderate and severe RSV infection, a Wilcoxon rank-sum test was used as the numbers were too small for parametric statistical tests. When protein mediators were measured by ELISA, any below the limit of detection (LOD) were assigned the lowest value of 12.5 pg/mL (R&D kits) or 6.25 pg/mL (Adipogen kit), and significant deviations from this value were measured using a Wilcoxon rank-sum test, assuming a hypothetical mean as the LOD. Confocal microscopy images were processed using FIJI ImageJ software.

## Results

### Patient demographics

Patients under the age of two years (*n* = 88) were recruited during hospitalisation with lower respiratory tract infections and bronchiolitis. Disease severity was based on peak hospitalisation, as previously described^9^; patients were classed as having severe infection (PICU admission, “Severe”, *n* = 40), or moderate (A&E/paediatric ward admission, “Moderate”, *n* = 48). The median duration of symptoms at the time of sample collection was 4 days for both groups. Those with severe infection were significantly younger (*P* = 0.044), lower weight (*P* = 0.018), had longer duration of hospitalisation (*P* < 0.0001) and higher prevalence of viral co-infections (*P* = 0.023). RSV was the most common respiratory pathogen amongst these patients, comprising 20 (49%) of those with severe infection and 23 (48%) of the moderate group.^9^ RNA from a total of 90 NPAs were analysed on the NanoString platform, and 57 of these passed QC criteria and were included in the analysis (Fig. E1). Of the patient samples included in the NanoString analysis, those with severe all-cause bronchiolitis exhibited a significantly lower weight (*P* = 0.015) and longer length of hospital stay (LOS; *P* < 0.0001). Similar to the larger cohort, there was a significantly higher prevalence of viral coinfections in the Severe group (*P* = 0.024) (Table E1). The viral coinfections were dominated by rhinovirus (44% (12/27) of the severe cohort, 17% (5/30) of the moderate cohort), followed by adenovirus, human metapneumovirus (HMPV), influenza A/B (IAV/IBV), or parainfluenza. There were a small number of bacterial co-infections in both cohorts (*n* = 4/27 in severe and 4/30 in moderate bronchiolitis). There were two cases of *Haemophilus influenzae* coinfection in the severe cohort, and the other bacterial coinfections were caused by *Pseudomonas aeruginosa*, *Bordatella pertussis, Enterobacter cloacae, Escherichia coli*, and coliform bacteria.

Healthy infants were not significantly younger than those with bronchiolitis, but there was a significant difference in the proportion of male:female infants (*P* = 0.008), higher weight at the time of recruitment (*P* = 0.018), and significantly less time spent in hospital (*P* < 0.0001). Six of the healthy infants, and 10/29 of the bronchiolitis group had a comorbidity spanning genetic disorders, jaundice and growth restriction, and chronic lung disease in the bronchiolitis group only. Whilst RSV was the most common pathogen in the bronchiolitis group (*n* = 15/27), bacterial and viral coinfections in the bronchiolitis group were mostly caused by rhinovirus (8/29, 28%), followed by *Escherichia coli, Staphylococcus aureus,* HMPV or adenovirus. As the healthy control group was recruited from those admitted for day surgery, they were ventilated for oxygen supplementation for the one day they were in hospital.

### Differential gene expression in severe vs moderate all-cause bronchiolitis

**Fig. 1:**
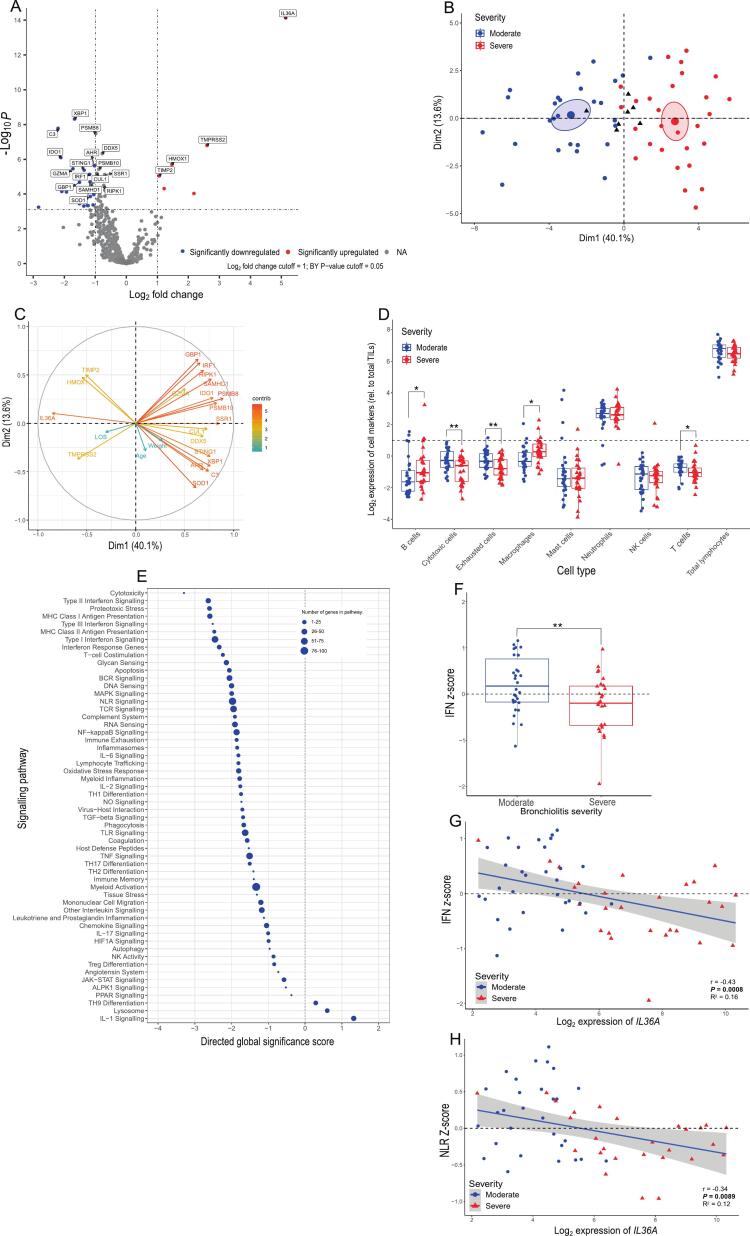
Deficient IFN signalling, and increased expression of *IL36A*, differentiate moderate and severe bronchiolitis. (A) Differential gene expression analysis in the NPAs of patients with severe bronchiolitis (*n* = 27) relative to moderate (*n* = 30). Genes are coloured depending on whether they were significantly upregulated (red) or significantly downregulated (blue) based on the Log_2_ fold change and adjusted P values after FDR correction in severe relative to moderate bronchiolitis. (B) FAMD analysis incorporating top 20 DEGs and quantitative clinical variables plotted by individuals, where patients are annotated by disease severity (red = severe, blue = moderate). Black triangles show qualitative variables. The ellipses represent 95% confidence intervals for each severity group. (C) Contributions of each measure to dimension 1 or 2 of the FAMD analysis. (D) Immune cell-associated markers measured in severe and moderate bronchiolitis. (E) Gene set enrichment analysis of pathways associated with severe bronchiolitis. Pathways were ordered based on their directed global significance scores and the size of the point represents the number of genes associated with each pathway. (F) IFN scores were generated for each patient by averaging z-scores for all genes relating to Type I-III IFN signalling in the NanoString host response panel (*n* = 82). G&H) Linear regression analysis between average IFN z-scores and *IL36A* expression, as well as between average NLR z-scores and *IL36A* expression, also confirmed by Spearman’s non-parametric correlation analysis. Points were coloured and shaped depending on the severity of all-cause bronchiolitis (blue circles = moderate, red triangles = severe). In panel A the cut-off values for the fold change and adjusted P values are 1 and 0.05, respectively. Adjusted P values were calculated using Benjamini-Yekutieli FDR correction. Statistical significance between groups in panels D and F were determined using Mann-Whitney tests. * = *P* < 0.05, ** = *P* < 0.01. ** = *P* < 0.01. ALPK1: ADP-heptose activates the protein kinase; BCR: B-cell receptor; contrib: contribution; DEGs: differentially expressed genes; HIF1A: hypoxia inducible factor 1 subunit alpha; JAK-STAT: janus kinase (JAK)-signal transducer and activator of transcription (STAT); LOS: length of hospital stay; MAPK: mitogen-activated protein kinase; MHC: major histocompatibility complex; NA: not applicable; NK: natural killer; NO: nitric oxide; NPAs; nasopharyngeal aspirates; NLR: NOD-like receptor; PPAR: peroxisome proliferator-activated receptors; TCR: T-cell receptor; TGF: transforming growth factor; TH1/2/9/17: Type 1/2/9/17 T helper; TILs: total infiltrating lymphocytes; TLR: Toll-like receptor; TNF: tumor necrosis factor; Treg: regulatory T cell. (For interpretation of the references to colour in this figure legend, the reader is referred to the web version of this article.)

Expression of 785 genes was analysed across the 57 samples. There were 57 significantly (Adj. *P* ≤ 0.05) differentially expressed genes (DEGs) in severe all-cause bronchiolitis relative to moderate. 51/57 DEGs showed reduced expression, including the transcription factor *XBP1*, as well as *C3, TIMP2, IDO1* and *GMZA*, and the interferon-induced chemokines *CXCL9* and *CXCL10*. There were six genes significantly upregulated in severe bronchiolitis relative to moderate, including the IL-1 family cytokines *IL36A* and *IL18*, along with *TMPRSS2*, *HMOX1*, *TIMP2*, and *CXCL5* (Fig. 1A, data file E1). We used the top 20 DEGs and quantitative clinical data (age, LOS, weight) to perform a FAMD analysis, showing separation of individuals based on severity and confirmed sensible clustering using the DEGs (Fig. 1B). The strongest contributors to this separation were DEGs including *IL36A* and *PSMB8* (Fig. 1C), while the patient demographics made weaker contributions to Dim1 and Dim2, suggesting age and weight of the patients were less strongly associated with disease severity. The main determinants (positive or negative) of Dim1 included *PSMB8*, *PSMB10*, *SSR1,* and *IL36A* (Fig. E2A). There was a strong association with all these genes and severity, suggesting that the downregulation of *PSMB8/10* and *SSR1* is most associated with disease severity, despite them not being the top downregulated DEGs in severe all-cause bronchiolitis (Fig. E2A). Other qualitative demographic factors were also checked for both separation and association with Dim1&2, but for sex, prematurity or coinfection, there was minimal separation or association with Dim1&2, further highlighting a lack of contribution of these clinical demographics to the severe bronchiolitis transcriptome (Fig. E2B–D).

Cell marker analysis showed a significant increase in markers associated with B cells (*P* = 0.036) and macrophages (*P* = 0.017), but a significant decrease in markers for cytotoxic cells (*P* = 0.005) and T cells (*P* = 0.041) in severe bronchiolitis, relative to moderate (Fig. 1D). Using gene set analysis, pathways were plotted based on their directed global significance scores (GSS) along with the number of genes associated with each pathway (Fig. 1E). There was a downregulation in pathways associated with Type I, II and III IFN signalling (GSS: −2.45, −2.62 and −2.5, respectively) and MHC class I and II antigen presentation (GSS: −2.6 and −2.5, respectively) in severe bronchiolitis, relative to moderate. A subset of genes associated with both interferon and MHC class I antigen presentation was associated with the immunoproteasome, with the genes encoding the three intracellular subunits (*PSMB8/9/10*) being significantly decreased in severe bronchiolitis (Adj. *P* = 0.000031, 0.022, and 0.0013, respectively). Only three pathways were upregulated in severe disease, of which IL-1 signalling was the strongest (GSS: 1.322) (Fig. 1E). Amongst the IL-1 signalling gene set, significantly elevated expression of *IL36A* (Log_2_ FC: 5.14; Adj. *P* = 2.69 × 10^−11^) and *IL18* (Log_2_ FC: 1.22; Adj. *P* = 0.008) were apparent. To better understand the differences in IFN signalling between disease severities, we generated average *z*-scores for each patient using all the genes in the Type I-III IFN pathways in the NanoString Host Response Panel (*n* = 82), and validated this approach using a published IFN-score panel,^27^ where we had corresponding genes (*n* = 23). There was no significant difference in *z*-scores between panels (*P* = 0.51, Fig. E3A), so the full NanoString panel was utilised for further analyses. The average IFN scores for those with moderate bronchiolitis were significantly higher than those with severe bronchiolitis (*P* = 0.009, Fig. 1F). Furthermore, there was a significant negative correlation with the average IFN scores and matched Log_2_ expression of *IL36A* (r = −0.43; *P* = 0.0008), confirmed by linear regression (Adj. R^2^ = 0.16; *P* = 0.002, Fig. 1G). There was no association between the IFN scores and *IL36G* expression by correlation (r = −0.24; *P* = 0.07, Fig. E3B) or linear regression (Adj. r^2^ = 0.034; *P* = 0.17). Another downregulated pathway of interest in severe bronchiolitis is the NLR signalling pathway (GSS: −1.98). As this pathway is important for activating the Type I IFN response and other innate immune responses,^28^ reduced pathogen sensing via NLRs could be one mechanism explaining the defective IFN response in severe infection. We observed a significant negative correlation between average NLR signalling scores and *IL36A* expression (r = −0.34; *P* = 0.0089), but not *IL36G* (r = −0.16; *P* = 0.23) (Figs. 1H and E3C, respectively). Together, these data indicated a broad depression in IFN signalling in severe bronchiolitis, relative to moderate, but a substantial increase in some IL-1 family cytokines, most notably IL-36α.

### Significant downregulation of the interferon signalling pathway in severe RSV

**Fig. 2:**
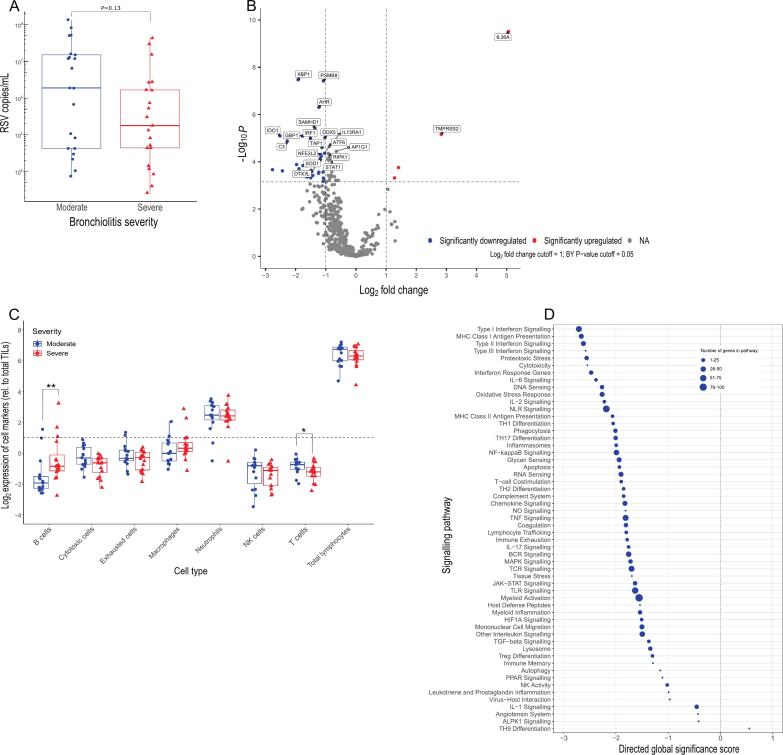
Reduced IFN expression and heightened *IL36A*, but not VL, in severe RSV bronchiolitis. (A) Viral load measured by qPCR in NPAs from patients with RSV (Severe n = 21, red triangles; Moderate *n* = 21, blue circles). (B) Differential gene expression analysis in severe (*n* = 13) relative to moderate (*n* = 14) RSV bronchiolitis. Genes are coloured depending on whether they were significantly upregulated (red) or significantly downregulated (blue) based on the Log_2_ fold change and adjusted P values after FDR correction in severe relative to moderate. (C) Immune cell-associated markers measured in severe and moderate RSV. (D) Gene set enrichment analysis of pathways associated with severe RSV, relative to moderate. Pathways were ordered based on their directed global significance scores and the size of the point represents the number of genes associated with each pathway. Table 1: Multiple linear regression of *IL36A* expression and clinical demographics. In panel B the cut-off values for the fold change and adjusted P values are 1 and 0.05, respectively. Adjusted P values were calculated using Benjamini-Yekutieli FDR correction. Statistical significance between groups in panel C was determined using Mann-Whitney test. * = *P* < 0.05, ** = *P* < 0.01. ALPK1: ADP-heptose activates the protein kinase; BCR: B-cell receptor; DEGs: differentially expressed genes; HIF1A: hypoxia inducible factor 1 subunit alpha; JAK-STAT: janus kinase (JAK)-signal transducer and activator of transcription (STAT); MAPK: mitogen-activated protein kinase; MHC: major histocompatibility complex; NA: not applicable; NK: natural killer; NO: nitric oxide; NLR: NOD-like receptor; NPAs; nasopharyngeal aspirates; PPAR: peroxisome proliferator-activated receptors; RSV: respiratory syncytial virus; TCR: T-cell receptor; TGF: transforming growth factor; TH1/2/9/17: Type 1/2/9/17T helper; TILs: total infiltrating lymphocytes; TLR: Toll-like receptor; TNF: tumor necrosis factor; Treg: regulatory T cell. (For interpretation of the references to colour in this figure legend, the reader is referred to the web version of this article.)

We next sought to determine if the weaker IFN response seen in severe all-cause bronchiolitis was similar in RSV specific disease, which was the most common pathogen in our cohort (Tables E1 and E2). RSV viral load in NPAs were analysed; 21 samples from severe infection and 21 from moderate were positive for RSV by qPCR. Although not statistically significant, viral load was higher in moderate RSV relative to severe (*P* = 0.13; Fig. 2A). In agreement with previous reports, this indicated that viral load is not a major determinant of RSV bronchiolitis severity.^9^, ^29^

Differential expression analysis identified 50 statistically significant (Adj. *P* < 0.05) differentially expressed genes between moderate (*n* = 14) and severe (*n* = 13) RSV cases (Table E2), there was significantly elevated expression of 4 genes: *IL36A, TMPRSS2, IL18,* and *HMOX1* in severe RSV (Fig. 2B). The genes that were significantly downregulated in severe RSV infection, relative to moderate (*n* = 46) included antiviral mediators spanning both chemokines (*CXCL9*, *CXCL11*) and interferon-stimulated genes (*IFI6, IFI16,* and *IRF1*). The elevated and decreased DEGs between severity groups in RSV bronchiolitis showed considerable overlap with the broader all-cause severe bronchiolitis group, suggesting their differential expression in severe bronchiolitis is not specific to RSV (Fig. E4). There were no pathogen-specific differences in the cell markers expressed in either severe or moderate RSV infection; the significant differences measured in B- and T cell markers were similar to that seen in the all-cause bronchiolitis group (Fig. 2C), but significance was lost for cytotoxic cells (*P* = 0.06), exhausted CD8 T cells (*P* = 0.32) and macrophages (*P* = 0.15). Gene set analysis showed similarities in pathways downregulated in severe RSV and the severe all-cause bronchiolitis analysis, including Type I and Type II interferon pathways and MHC class I antigen presentation (GSS: −2.13, −2.16, −2.1, respectively; Fig. 2D, Fig. E5A–C). There was no significant difference in the average IFN *z*-scores in the larger all-cause bronchiolitis group compared to those with confirmed RSV infection (*P* = 0.15, Fig. E6A). These data suggested that reduced IFN signalling and enhanced *IL36A* expression are features of severe bronchiolitis common to different pathogens. We also found weak, non-significant, correlations between IFN score and VL (r = 0.23; *P* = 0.29), and between *IL36A* expression and VL (r = −0.34; *P* = 0.11) (Fig. E6B&C, respectively), further highlighting that RSV viral load does not seem to play a role in the immune features of severe RSV disease. To identify whether there were any confounding factors associated with IL-36 production in our cohort, multiple linear regression analysis was performed to relate *IL36A* levels to clinical data (Table 1). This showed a significant positive association between the expression of *IL36A* and the length of hospital stay (T = 5.69, Pr(>|t|) = 0.0000012) after accounting for other variables, but no correlation with other clinical demographics included in the analysis such as age, weight or bacterial/viral co-infections.

**Table 1:** Multiple linear regression of *IL36A* expression in nasopharyngeal aspirates and clinical demographics.

Coefficients:	Estimate Std.	Error	T value	Pr(>[t])	Significant?
(Intercept)	5.69	0.73	7.85	1.09e-09	***
**Length of hospital stay**	**0.26**	**0.045**	**5.69**	**1.19e-06**	***
Symptoms to sample (days)	−0.015	0.014	−1.053	0.30	NS
Age (weeks)	−0.0058	0.0081	−0.72	0.48	NS
Bacterial coinfection (Y/N)	−0.096	0.56	−0.17	0.86	NS
Viral coinfection (Y/N)	0.24	0.43	0.56	0.58	NS
Sex (M/F)	−0.013	0.43	−0.03	0.98	NS

### IL-36 is elevated in severe bronchiolitis

**Fig. 3:**
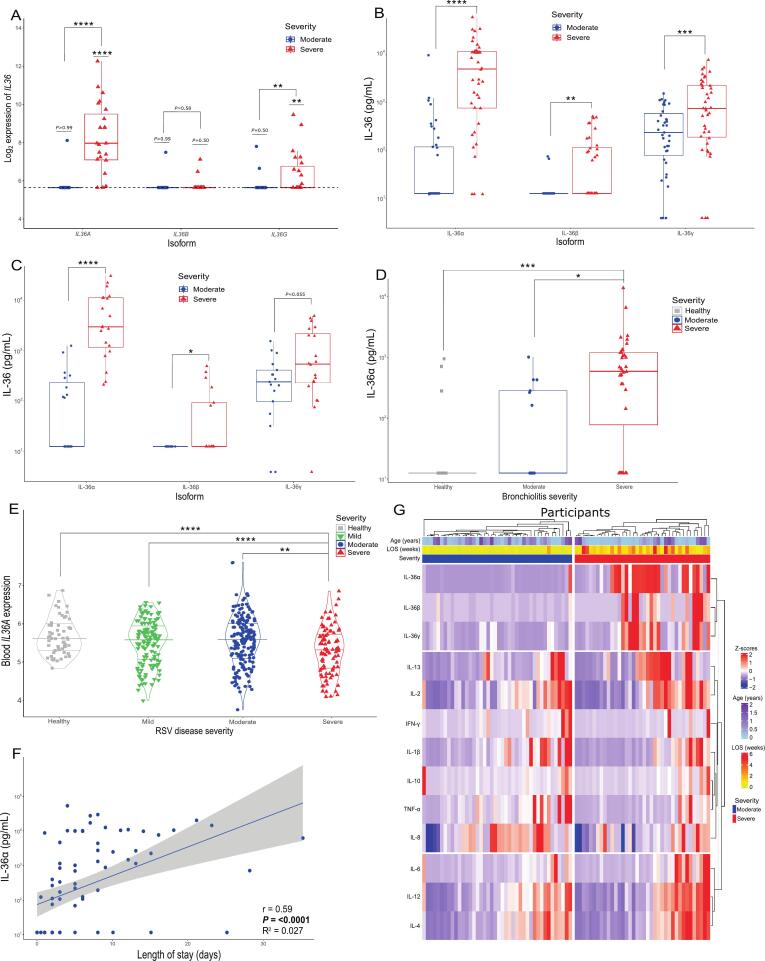
IL-36 is elevated in severe bronchiolitis only and is positively correlated with the length of hospitalisation. A) Expression of *IL36A*, *IL36B*, and *IL36G* in moderate *(n* = 21) and severe *(n* = 21) bronchiolitis*,* in RNA isolated from NPAs, and measured by NanoString. B) IL-36 protein levels in nasopharyngeal aspirates from either moderate (*n* = 48) or severe (*n* = 40) bronchiolitis. C) IL-36 protein levels in nasopharyngeal aspirates (RSV + group) from either moderate (n = 20) or severe (n = 20) RSV infection. D) Protein concentrations of IL-36α in nasosorption samples from healthy controls (grey squares; n = 15), moderate bronchiolitis (blue circles; n = 13) or severe bronchiolitis (red triangles; n = 36). E) Gene expression of *IL36A* was measured in blood using bulk transcriptomics in healthy infants (*n* = 56), or infants with mild (*n* = 72), moderate (*n* = 92) or severe (*n* = 48) RSV infection (https://doi.org/10.1002/ctm2.1507). (F) Spearman’s non-parametric correlation and simple linear regression between IL and 36α concentration and the length of hospitalisation. (G) Heatmap of Z-scores of 13 protein mediators measured in NPAs. Both rows and columns were split by K-means clustering. Patients are annotated with peak severity (blue = moderate, red = severe), age, and length of hospital stay (LOS). In panel A the line at y = 5.36 represents the threshold for gene expression after background thresholding, A chi-squared test in panel A determined significant increase in expression above the background threshold, and Mann-Whitney test determined significance between severe and moderate infection in panels A–C. * = *P* < 0.05; ** = *P* < 0.01; *** = *P* < 0.001; **** = *P* < 0.001. (For interpretation of the references to colour in this figure legend, the reader is referred to the web version of this article.)

As the top DEG in our analysis was the elevation of *IL36A* in severe bronchiolitis*,* we studied the expression of all IL-36 isoforms. One-sample T-tests demonstrated that expression of *IL36A/B/G* in moderate samples did not differ from the lower limit of sensitivity (all *P* > 0.05), demonstrating that *IL36* gene expression is not evident in moderate bronchiolitis. By contrast *IL36A* and *IL36G* were detected in severe cases and were significantly raised relative to moderate (*P* < 0.0001 and *P* = 0.0034, respectively, Fig. 3A). We next sought to confirm the elevation of IL-36 in NPAs at the protein level, by ELISA. The levels of IL-36α, IL-36β, and IL-36γ were all significantly higher in severe all-bronchiolitis compared to moderate (*P* < 0.0001; *P* = 0.0002; *P* = 0.002, respectively), with the difference between severity groups most pronounced for IL-36α (Fig. 3B). There was a significant positive correlation between the protein levels of IL-36α and the gene expression of *IL36A* (r = 0.65, *P* < 0.0001; Fig. E7A).

Heightened levels of IL-36 were also evident in patients with RSV bronchiolitis, though differences in IL-36γ between severity groups were not significant (*P* = 0.055; Fig. 3C). The significant increase in IL-36α was also not dependent on the amount of total protein measured in the neat NPAs, as differences were still significant after normalisation to total protein (Fig. E7B). To enable a comparison to levels in healthy infants, we next measured the concentration of IL-36α in nasosorption samples from healthy controls (*n* = 15), moderate bronchiolitis (*n* = 13) and severe bronchiolitis (*n* = 36), combining samples from both RSVSAM and ELLI studies (Tables E1–E3). The levels of IL-36α in severe bronchiolitis were significantly higher than in healthy controls (*P* = 0.0007), and moderate bronchiolitis (*P* = 0.002) (Fig. 3D), indicating that upper airway IL-36 is a characteristic marker of severe bronchiolitis less evident in milder forms of disease. Comparing the levels of IL-36α in matched samples from the upper (NPA) and lower (BAsp) airways revealed that levels of IL-36α were typically higher in the upper airway, relative to the lower airway, in those with severe all-cause bronchiolitis (Fig. E7C, P = 0.018, n = 15). Furthermore, there was a significant negative correlation between matched NPA and BAsp IL-36α levels (r = −0.64; *P* = 0.013), though this was largely driven by 3 BASP samples with high IL-36α levels (Fig. E7D), suggesting that immune responses in the upper airway may not necessarily directly replicate those in the lower airway. We next sought to determine whether IL-36 levels in the blood were similarly associated with RSV disease severity. Using whole blood transcriptome data from a previously reported cohort of infants with RSV,^30^ we observed that *IL36A* expression was lower in severe RSV cases than in either mild or moderate forms of disease, or in healthy controls (all *P* < 0.05, Fig. 3E). Together, these data indicated that the elevation in upper airway IL-36 levels associated with disease severity were not necessarily reflective of lower airway inflammation, or evident in the peripheral blood.

We next sought to understand if differences in IL-36α levels between severity groups were associated with clinical differences. NPA levels of IL-36α correlated with length of hospital stay (r = 0.59, *P* < 0.0001, Fig. 3F) but not with other measures of disease severity, such as paediatric risk of mortality (PRISM) score^31^ (r = −0.013, *P* = 0.99) or days on invasive ventilation (severe group only) (r = −0.20, *P* = 0.34; Fig. E7E&F). These data indicated that elevation of *IL36A* expression is associated with bronchiolitis severity, independent of demographic differences between groups. We then looked at the protein concentration of IL-36 isoforms in the context of other protein mediators measured in matched NPAs (Fig. 3G). *Z*-scores were generated for each mediator to account for differences in quantities between mediators. Mediators were hierarchically clustered, which grouped all IL-36 isoforms together and further highlighted the absence of these proteins in patients with moderate disease.

### RSV infection of HNEC induces both IFNs and IL-36

Epithelial cells have been reported to secrete IL-36,^32^ so we next sought to determine whether IL-36α production was a feature of *in vitro* RSV infections of human nasal epithelial cells (HNEC). For the generation of HNEC cultures, three healthy volunteers were recruited, and epithelial cells collected via nasal brushings. All volunteers were aged between 18 and 25 and did not have any respiratory morbidities, including asthma or infection. Basal cells from nasal brushings were plated on Transwells and grown under ALI conditions to produce a 3D, well-differentiated, epithelium.

**Fig. 4:**
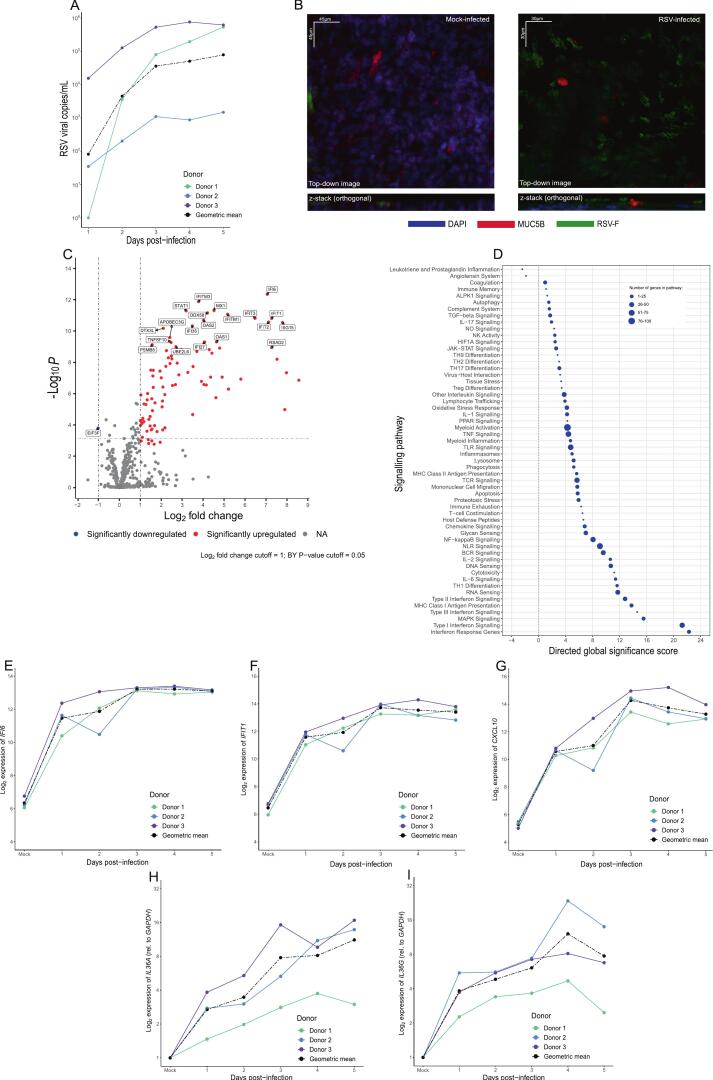
RSV-infection induces strong antiviral responses, as well as *IL36A* expression, *in vitro.* HNEC cultures were infected with RSV A2 at an MOI of 0.1; apical washes were collected at each day post-infection (DPI). (A) RSV copies measured in the apical washes of infected HNEC cultures, quantified by qPCR. (B) Confocal microscopy of mock- and RSV-infected cultures at 1DPI and stained for nuclei (DAPI, blue), mucus-producing cells (MUC5B, red) and RSV-infected cells (RSV-F, green). (C) Differential gene expression analysis in RSV-infected HNECs relative to mock-infected HNECs at 5DPI (n = 3 donors). Genes are coloured depending on whether they were significantly upregulated (red) or downregulated (blue) based on the Log2 fold change and adjusted P values after FDR correction. The cut-off values for the fold change and adjusted P values are 1 and 0.05, respectively. Adjusted P values were calculated using Benjamini-Yekutieli FDR correction. (D) Gene set enrichment analysis of pathways associated with RSV-infected HNECs, relative to mock-infected (*n* = 3 donors) at 5DPI. Pathways were ordered based on their directed global significance scores and the size of the point represents the number of genes associated with each pathway. Expression of (E) *IFI6*, (F) *IFIT1*, (G) *CXCL10*, (H) *IL36A*, and (I) *IL36G,* measured using NanoString or qPCR, in RSV-infected ALI cultures at each DPI, relative to uninfected cultures. The black dotted line denotes the geometric mean from the three donors. In panels A and E-I, results are plotted for each donor alongside a geometric mean of all donors (black). ALPK1: ADP-heptose activates the protein kinase; BCR: B-cell receptor; DEGs: differentially expressed genes; HIF1A: hypoxia inducible factor 1 subunit alpha; JAK-STAT: janus kinase (JAK)-signal transducer and activator of transcription (STAT); MAPK: mitogen-activated protein kinase; MHC: major histocompatibility complex; NA: not applicable; NK: natural killer; NO: nitric oxide; NLR: NOD-like receptor; NPAs: nasopharyngeal aspirates; PPAR: peroxisome proliferator-activated receptors; RSV: respiratory syncytial virus; TCR: T-cell receptor; TGF: transforming growth factor; TH1/2/9/17: Type 1/2/9/17 T helper; TILs: total infiltrating lymphocytes; TLR: Toll-like receptor; TNF: tumor necrosis factor; Treg: regulatory T cell. (For interpretation of the references to colour in this figure legend, the reader is referred to the web version of this article.)

HNEC cultures were infected with RSV A2 at an MOI of 0.1, and the apical washes were collected at each day post infection (DPI). Evidence of replicating virus was seen as increased RSV viral loads from 1 to 2DPI. There were differences in the scale of viral shedding between donors, but all plateaued around 3DPI (Fig. 4A). Confocal microscopy showed a strong staining of DAPI (blue) across the culture and evidence for mucus-producing cells within the culture, as shown by the staining of MUC5B (red). RSV-F protein (green) was predominantly localised within ciliated epithelial cells, in line with the susceptibility of these cells to RSV infection (Fig. 4B). The presence of diverse epithelial cell types were also confirmed by qPCR of a set of 6 characteristic genes. All six genes were expressed in mock and infected cultures, with a downregulation of *FOXJ1* and *PIGR* in RSV-infected cultures by 5DPI (Fig. E8A–F). Transcriptomic analysis revealed a robust interferon-based response to infection at 5DPI (Fig. 4C), marked by significant expression of *IFI6/27/35* and *IFIT1-3* (DEGs for 1-5DPI in data file E2). Pathway analysis further showed this robust antiviral response, with IFN response genes and Type I-III IFN signalling pathways having high directed GSS: 22.3, 21.3, 12.8 and 14.5, respectively (Fig. 4D). Expression of the prototypical antiviral mediators *IFI6, IFIT1,* and *CXCL10* increased as early as 1DPI and remained elevated throughout infection (Fig. 4E–G, respectively). The increased production of CXCL10 was further confirmed by ELISA, using basolateral supernatants from both RSV- and mock-infected HNEC cultures. Although CXCL10 was undetectable in the mock-infected cultures, an increase in the concentration was measured across all three RSV-infected HNEC cultures throughout infection (Fig. E9). While they were not detected in NanoString analysis, qPCR observed increased *IL36A* and *IL36G* expression throughout infection (Fig. 4H&I, respectively). Interestingly, despite the increased expression of *IL36A* and *IL36G* in RSV-infected cultures, neither isoform was detectable in apical secretions or basolateral compartment at the protein level. These data indicate that IL-36 is expressed following RSV infection of HNECs but is not secreted at a quantifiable level.

### Soluble factors in NPAs induce IL-36

**Fig. 5:**
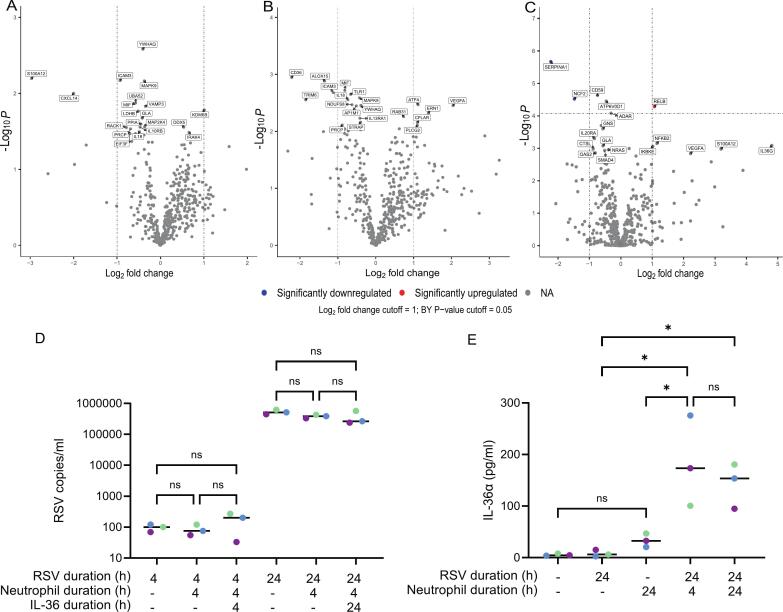
Neutrophils trigger the release of mucosal IL-36α *in vitro*. Differential gene expression analysis in human nasal epithelial cell (HNEC) cultures after exposure to A) LPS relative to mock, B) rIL-17A relative to mock, or C) NPA supernatants relative to mock. Genes are coloured depending on whether they were significantly upregulated (red) or downregulated (blue), or not significant (grey) based on the Log_2_ fold change and adjusted P values after FDR correction. The cut-off values for the log2 fold change and Benjamini-Yekutieli FDR adjusted P values are 1 and 0.05, respectively. To determine the effect of neutrophils on RSV replication and IL-36 release, inverted HNECs were cultured at air–liquid interface and infected with RSV for 4 h or 24 h, with or without the addition of basolateral neutrophils or recombinant IL-36. D) RSV was measured by RT-qPCR at 4 h or 24 h post-infection of HNECs in culture lysates, in the absence or presence of basolateral neutrophils (for the final 4 h of the infection) or recombinant IL-36. E) IL-36α concentration in the apical washes of HNECs 24 h after infection with RSV, in the absence or presence of basolateral neutrophils. Neutrophils were added either at the time of infection, or 20 h after infection was initiated. In panels D and E, dots are coloured based on HNEC donor (mean of technical duplicates, *n* = 3) alongside the median of all donors (black line). Statistical significance in panels D and E were measured using ANOVA with Tukey’s correction for multiple comparisons. * = *P* < 0.05. (For interpretation of the references to colour in this figure legend, the reader is referred to the web version of this article.)

These *in vitro* studies thus far indicated a strongly IFN driven response to RSV infection, reminiscent of the IFN-led response evident in moderate bronchiolitis, that is absent in severe disease. We next investigated whether alterations to the HNEC culture environment could perturb this IFN-led response to be more similar to that seen in severe bronchiolitis. A previous study has shown an induction of IL-36 in cell culture models by IL-17, LPS, or stimulation by dsRNA.^32^ As we previously showed heightened IL-17 in severe RSV,^9^ we first studied whether IL-17 could drive the strong IL-36 expression observed in our severe bronchiolitis cohort. HNEC cultures were incubated with basolateral rIL-17A or LPS in the apical compartment, with or without RSV infection. Transcriptomic analysis revealed no significant DEGs between the RSV-infected cultures and those stimulated with LPS or rIL-17A alongside RSV infection (Fig. 5A & B, respectively). rIL-17A and LPS therefore did not alter the response of these cultures to RSV.

Given that we could not induce *IL36A* using non-viral stimulants, we then looked at whether ligands present in primary human NPA samples from RSV infected infants could stimulate IL-36 release in the 3D HNEC cultures. Six cell-free NPAs were used to stimulate the HNEC cultures, and gene expression was measured relative to mock-infected. *SERPINA1* (Log_2_ FC = −2.23, Adj. *P* = 0.0067) and *NCF2* (Log_2_ FC = −1.48, Adj. *P* = 0.028) were significantly downregulated in cultures stimulated with NPAs from patients with severe bronchiolitis, relative to mock-infected (Fig. 5C). *RELB*, a transcription factor governing the non-canonical pathway to NF-kB activation^33^ was the only significantly upregulated DEG in NPA-treated cultures. Interestingly, *IL36G* was upregulated in NPA-treated cultures, but missed statistical significance following FDR correction (Log_2_ FC = 4.81, Adj. *P* = 0.053, unadjusted *P* = 0.00022, Fig. 5C). However, *IL36A* was removed from the analysis following background thresholding, suggesting little expression and no upregulation following NPA stimulation. There were no significant increases in the expression of *IL36A* or *IL36B* in cultures treated with NPAs, or NPAs and RSV, relative to mock (Figs. E10A & B). IL-36 could not be detected at a protein level in these experiments. The expression of *IL36G* was significantly higher in the cultures that received either treatment and the levels were significantly enhanced by the additional RSV infection, compared to the cultures that received NPA only. However, expression was still highest in the cultures that were infected with RSV alone.

### Neutrophils amplify IL36A release from RSV infected HNEC cultures

Given the ability of NPAs to weakly induce *IL36A* and *IL36G* expression in HNECs, and the role of neutrophil-derived proteases in cleaving IL-36 to biological maturity,^34^, ^35^ we reasoned that soluble factors from neutrophils may amplify IL-36 secretion by epithelial cells. Neutrophils were added to the basolateral side of inverted HNEC cultures, using transwell inserts with 0.4 µm pores to prevent neutrophil migration to the apical epithelial layer. RSV was detectable in HNEC lysates 4 h after infection, with viral load increasing by 24 h. Neither the addition of neutrophils, nor neutrophils with recombinant (r)IL-36, impacted the viral load in HNEC lysates (Fig. 5D). IL-36α secretion was not significantly induced in the RSV-alone cultures after 24 h (median 4.63 pg/ml), relative to mock (median 6.08 pg/ml). HNECs cultured with basolateral neutrophils for 24 h did secrete more IL-36α into the apical fluid (median 32.59 pg/ml) but this was not significant (*P* = 0.94). However, IL-36α secretion was significantly higher in RSV infected HNECs cultured with neutrophils for 4 h (median 173.41 pg/ml, *P* = 0.011 relative to RSV-alone, Fig. 5E). IL-36α secretion was not further enhanced when neutrophil co-cultures were extended to 24 h (median 153.67 pg/ml). TEER readings remained unchanged during these 24 h cultures. Together, these data demonstrate that neither neutrophils nor rIL-36 disrupt RSV entry or replication but that neutrophils alone can trigger IL-36α release from HNECs, which is enhanced in the presence of RSV.

## Discussion

We demonstrate that elevated mucosal IL-36α is a marker of severe paediatric bronchiolitis, irrespective of cause, and that its production during RSV infection of nasal epithelial cells is enhanced by neutrophils. Severe bronchiolitis was also marked by diminished interferon-driven responses and equivalent or lower viral load than observed in milder disease.

Our understanding of the immunological mechanisms active during life-threatening paediatric bronchiolitis has improved, but there is still uncertainty on which features of the immune response contribute to pathology in the respiratory mucosa. In this study, we utilised transcriptomics to provide a broad view of the markers and pathways that differentiate paediatric patients with severe versus moderate bronchiolitis. We found an overall decrease in the expression of interferon signalling pathways in severe bronchiolitis, including in PCR confirmed RSV infection, in agreement with other reports.^9^, ^10^, ^36^ Pathways of particular interest in severe RSV infection include the downregulation of genes associated with the 20S core of the immunoproteasome (*PSMB8/9/10*), which is vital for processing viral antigens and presenting them via MHC class I, driving the CD8 + T cell-mediated killing of pathogen-infected cells.^37^ Also contributing to this pathway of antigen processing is TAP-1, which works alongside TAP-2 to facilitate the transport of processed antigens into the endoplasmic reticulum.^37^ *TAP1* was significantly downregulated in severe RSV disease, indicating a reduction in this antigen processing pathway. This pathway is induced by IFN-γ, suggesting a potential downstream effect of reduced interferon signalling on the adaptive immune response during severe RSV infection. We also observed downregulation of genes associated with the NLRP3 inflammasome (*APP*, *CARD16*, *CARD17*, *CASP1*, *NLRP3*). Although not used for processing IL-36α, the inflammasome is important for processing other IL-1 cytokines, including IL-18.^38^ *IL18* was upregulated in severe disease, indicating elevated expression of this cytokine despite diminished ability to process it to biological maturity. Furthermore, we measured a significant increase in expression of *HMOX1*, which has been shown to inhibit the NLRP3 inflammasome and reduce the production of IL-1 cytokines via this pathway.^39^, ^40^ The reduction of *TLR4* expression in our analysis may be indicative of reduced pathogen- or damage- associated molecular pattern sensing in severe infection, which could lead to decreased activation of the NLRP3 signalling pathway.^41^

While most DEGs were downregulated in severe infection relative to moderate, the upregulated DEGs provided particular immunological insights. *TMPRSS2*, which is indispensable for SARS-CoV-2 entry into cells,^42^ was elevated in severe infection. The scale of *IL36A* upregulation in the upper airway in severe disease was particularly high, while our analysis of data from a previously published cohort^30^ demonstrated that *IL36A* expression was actually decreased in the whole blood during severe RSV. The human protein atlas indicates that *IL36A* expression in PBMC is restricted to T cells, so the decreased blood *IL36A* expression observed in our analysis may reflect blood lymphopenia commonly observed during severe disease.^43^ This difference between the upper airway and blood highlights the importance of understanding mucosal inflammation in severe bronchiolitis.

IL-36 exists as three isoforms, −α, −β and −γ, within the family of IL-1 cytokines; full-length proteins require proteolytic processing for biological activity.^35^ Unlike other IL-1 cytokines, their cleavage is not dependent on the NLRP3 inflammasome, but instead on neutrophil-derived proteases.^35^ This is a particularly intriguing association given the abundance of neutrophils in the airway during bronchiolitis.^51^ This suggests that the cellular environment in bronchiolitis may be suitable for neutrophil-mediated activation of epithelial cell secreted IL-36. The biology of IL-36 cytokines has mostly been investigated in the context of psoriasis and atopic dermatitis where it promotes inflammation, while recent studies have shown that IL-36α and IL-36γ can promote proinflammatory responses through both chemoattraction and activation of neutrophils.^34^, ^44^ IL-36α and −β in the sputum is able to differentiate cohorts of asthma based on their immunological phenotype; sputum IL-36 was more closely associated with neutrophilic asthma, as opposed to eosinophilic or paucigranulocytic asthma.^45^ This, combined with the positive association of IL-36 and the number of neutrophils, further suggests a role of IL-36 in neutrophil-mediated airway inflammation in asthma.^45^ Upon binding to the IL-36 receptor (IL-36R), IL-36α and IL-36γ have the potential to induce IL-1 family cytokines, but also IL-36 itself, forming a positive feedback loop.^46^ Other IL-1 cytokines can also promote a more inflammatory environment and the production of mucus during viral infection, as shown by the reduction in both following treatment with the IL-1 receptor antagonist, IL-1Ra.^47^ In IL-36R-deficient mice, exposure to cigarette smoke or infection with H1N1 IAV led to reduced neutrophil-driven inflammation and airway damage compared to controls, further highlighting how IL-36 can contribute to epithelial damage during infection or exposure to pollutants. Increased IL-36 could therefore also contribute to the enhanced airway inflammation and damage seen in severe bronchiolitis.^34^ These data indicate that IL-36 neutralisation could be a target for decreasing pathological inflammation in severe bronchiolitis. Crucially, monoclonal IL-36 receptor antagonists have already been developed and are in clinical use for generalized pustular psoriasis.^48^ Furthermore, a small-molecule antagonist of the IL-36 receptor was recently described,^49^ suggesting that this pathway may be amenable to therapeutic manipulation. Further characterisation of the function of mucosal IL-36 in severe bronchiolitis is warranted before trials of these therapies could be considered.

With the role of IL-36 in amplifying neutrophilic inflammation, and the strong *IL36A* signature in our analysis, we validated the transcript data by measuring IL-36 isoforms at the protein level. This confirmed increased IL-36α levels in severe infection, in addition to heightened IL-36γ. In the human RSV challenge model, we previously showed that participants who developed symptomatic RSV infection post-inoculation had mucosal neutrophilic inflammation evident pre-challenge, compared to those that resisted infection, with one of the genes associated with this susceptibility being *IL36G.*^50^ These results lead to the question of whether mucosal neutrophilia and IL-36-led inflammation may precede infection in infants with severe bronchiolitis.

Using HNEC cultures grown under ALI conditions, we observed an induction of both *IL36A* and *IL36G* in RSV-infected cultures. However, IL-36 secretion was not evident until neutrophils were present in co-culture, indicating that neutrophil derived soluble factors may be essential for IL-36 release from infected epithelial cells. Protein measurements of IL-36 secretion after *in vitro* infection in the absence of neutrophils were largely close to the lower limit of detection of the assay, so the degree of correlation between gene expression and protein production could not be reliably assessed. We also used NPA samples to treat HNECs, which induced expression of *IL36G,* though this was not statistically significant after FDR correction. This suggests that mucosal mediators present in bronchiolitis respiratory secretions may induce *IL36* directly. However, it could still not be detected at the protein level in apical washes. A study using bacteria and fungi to infect cell culture models found a similar result, where IL-36 could only be detected in the cell lysate and not cell supernatant.^51^ Our data suggests that although RSV can induce *IL36A*, its production and release into the respiratory mucosa, as observed in severe bronchiolitis, may be dependent on the presence of products secreted by neutrophils.

Neutrophils are suggested to be key players in paediatric RSV bronchiolitis, representing ∼ 85% of the total cell count in the bronchoalveolar lavage of hospitalised patients.^52^ They are vital immune cells in the initial antiviral response, but their high abundance in the airways can contribute to epithelial damage during infection.^53^ This has been particularly well characterised *in vitro,* where the interaction between neutrophils and airway epithelial cells during RSV infection was modelled.^18^ RSV infection was associated with increased expression of activation markers on co-cultured neutrophils, as well as the generation of clusters of neutrophils that adhere to RSV-infected cells. Although important for combatting infection, these mechanisms may also be indicative of neutrophil-mediated immunopathology and epithelial damage during severe RSV infection, that may be mediated in part by IL-36.

Our study has limitations. Firstly, most of our analysis is based on samples from the upper airways, which may differ from lower respiratory inflammation. For the analysis, we used a semi-targeted transcriptomics approach which measured the expression of 785 genes, rather than whole-transcriptome approaches. Advancing this analysis with bulk or single-cell RNA-sequencing would enable additional insights but is limited by the integrity of RNA that can be extracted from such samples. Due to the bulk RNA nature of our analyses, we could not distinguish whether cell-marker results are due to differences in the frequencies of these cells in the airways, or differences in the transcriptional activity of an equivalent number of cells. The panel we used spans innate and adaptive immune responses, giving an overview of most immune pathways. Furthermore, our method of NPA collection cannot be conducted on the dry upper airways of healthy infants, so nasosorption sampling was instead used to enable collection of samples from healthy controls and validate key observations at the protein level. Next, the use of HNECs from healthy adults limits the mechanistic work for modelling RSV infection, particularly as we measured a strong interferon-based response which highlights disparity between these cultures and severe paediatric infection. Reports have varied on whether adult and paediatric HNECs differ in their response to viral infection,^54^, ^55^ so our results in HNECs derived from adult donors could differ from those derived from children. HNECs derived from young children have been reported; however, while collecting such invasive samples from neonates, to match the age of infants in our cohort, may provide additional insights it presents considerable logistic and ethical challenges. Additionally, studies have utilised neutrophils collected from paediatric volunteers for epithelial-neutrophil co-culture and showed they are more activated and apoptotic in response to RSV infection, compared to adult-derived neutrophils.^56^

## Conclusions

In summary, elevated IL-36 levels in the mucosa, but not blood, are associated with severity during paediatric bronchiolitis, alongside a weakened interferon response, relative to milder infections. IL-36 was secreted by epithelial cells during *in vitro* RSV infections, a process enhanced by the presence of neutrophils in co-culture. IL-36 antagonists are in clinical use to treat inflammatory skin conditions, warranting further study of the function of mucosal IL-36 and the possibility of therapeutic intervention in paediatric bronchiolitis.

## CRediT authorship contribution statement

**Megan V.C. Barnes:** Writing – review & editing, Writing – original draft, Visualization, Methodology, Investigation, Formal analysis, Data curation. **Sophie W.H. Stretch:** Writing – original draft, Visualization, Methodology, Investigation, Formal analysis. **Dawid Swieboda:** Visualization, Investigation. **Claudia Efstathiou:** Visualization, Methodology, Investigation. **Fiona J. Culley:** Methodology, Investigation, Funding acquisition. **Trevor T. Hansel:** Supervision, Methodology, Investigation, Funding acquisition, Conceptualization. **Sam Strickson:** Supervision, Methodology, Investigation. **Xavier Romero-Ros:** Supervision, Methodology, Investigation. **E. Suzanne Cohen:** Methodology, Investigation, Funding acquisition. **Simon Nadel:** Writing – original draft, Supervision, Resources, Methodology, Investigation, Funding acquisition, Data curation, Conceptualization. **Peter J.M. Openshaw:** Writing – review & editing, Writing – original draft, Supervision, Resources, Investigation, Funding acquisition, Conceptualization. **Ryan S. Thwaites:** Writing – review & editing, Writing – original draft, Visualization, Supervision, Funding acquisition, Formal analysis, Data curation, Conceptualization.

## Declaration of competing interest

The authors declare the following financial interests/personal relationships which may be considered as potential competing interests: Peter Openshaw reports financial support was provided by National Institute for Health Research. Ryan Thwaites reports financial support was provided by Imperial Biomedical Research Centre. Ryan Thwaites reports financial support was provided by Health Protection Research Unit (HPRU) in Respiratory Infections at Imperial College London. Megan Barnes reports financial support was provided by Biotechnology and Biological Sciences Research Council. Ryan Thwaites reports financial support was provided by MRC and Asthma UK Centre in Allergic Mechanisms of Asthma. Dawid Swieboda reports a relationship with AstraZeneca that includes: employment. Sam Strickson reports a relationship with AstraZeneca that includes: employment. Xavier Romero-Ros reports a relationship with AstraZeneca that includes: employment. E. Suzanne Cohen reports a relationship with AstraZeneca that includes: employment. Fiona Culley reports a relationship with Sanofi that includes: consulting or advisory. Peter Openshaw reports a relationship with GSK that includes: consulting or advisory and speaking and lecture fees. Peter Openshaw reports a relationship with Moderna Inc that includes: consulting or advisory and speaking and lecture fees. Peter Openshaw reports a relationship with Janssen Pharmaceuticals Inc that includes: consulting or advisory and speaking and lecture fees. Peter Openshaw reports a relationship with Seqirus Srl that includes: consulting or advisory and speaking and lecture fees. Peter Openshaw reports a relationship with Pfizer that includes: consulting or advisory and speaking and lecture fees. Peter Openshaw reports a relationship with AstraZeneca that includes: consulting or advisory and speaking and lecture fees. Peter Openshaw reports a relationship with Shionogi Inc that includes: consulting or advisory and speaking and lecture fees. Peter Openshaw reports a relationship with Bionet VN that includes: consulting or advisory and speaking and lecture fees. Peter Openshaw reports a relationship with Sanofi that includes: consulting or advisory and speaking and lecture fees. Ryan Thwaites reports a relationship with Sanofi that includes: speaking and lecture fees. Ryan Thwaites reports a relationship with AstraZeneca that includes: consulting or advisory and speaking and lecture fees. Ryan Thwaites reports a relationship with Moderna Inc that includes: consulting or advisory. If there are other authors, they declare that they have no known competing financial interests or personal relationships that could have appeared to influence the work reported in this paper.
