# Supplementary figures and images for: Mucosal IL-36 is a defining feature of severe paediatric bronchiolitis
Source: Mucosal Immunol. 2026 Apr;19(2):1907–21. doi: 10.1016/j.mucimm.2026.01.012 (PMC13195398; doi:10.1016/j.mucimm.2026.01.012)

Figure S1

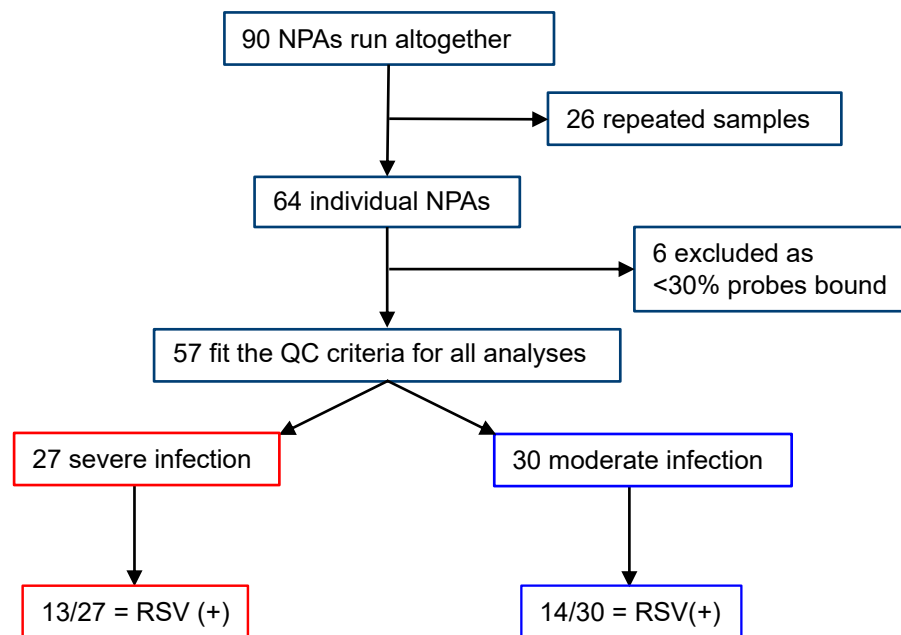

Supplement: Supplementary Data 1 [file mmc1.pdf]

Figure S2

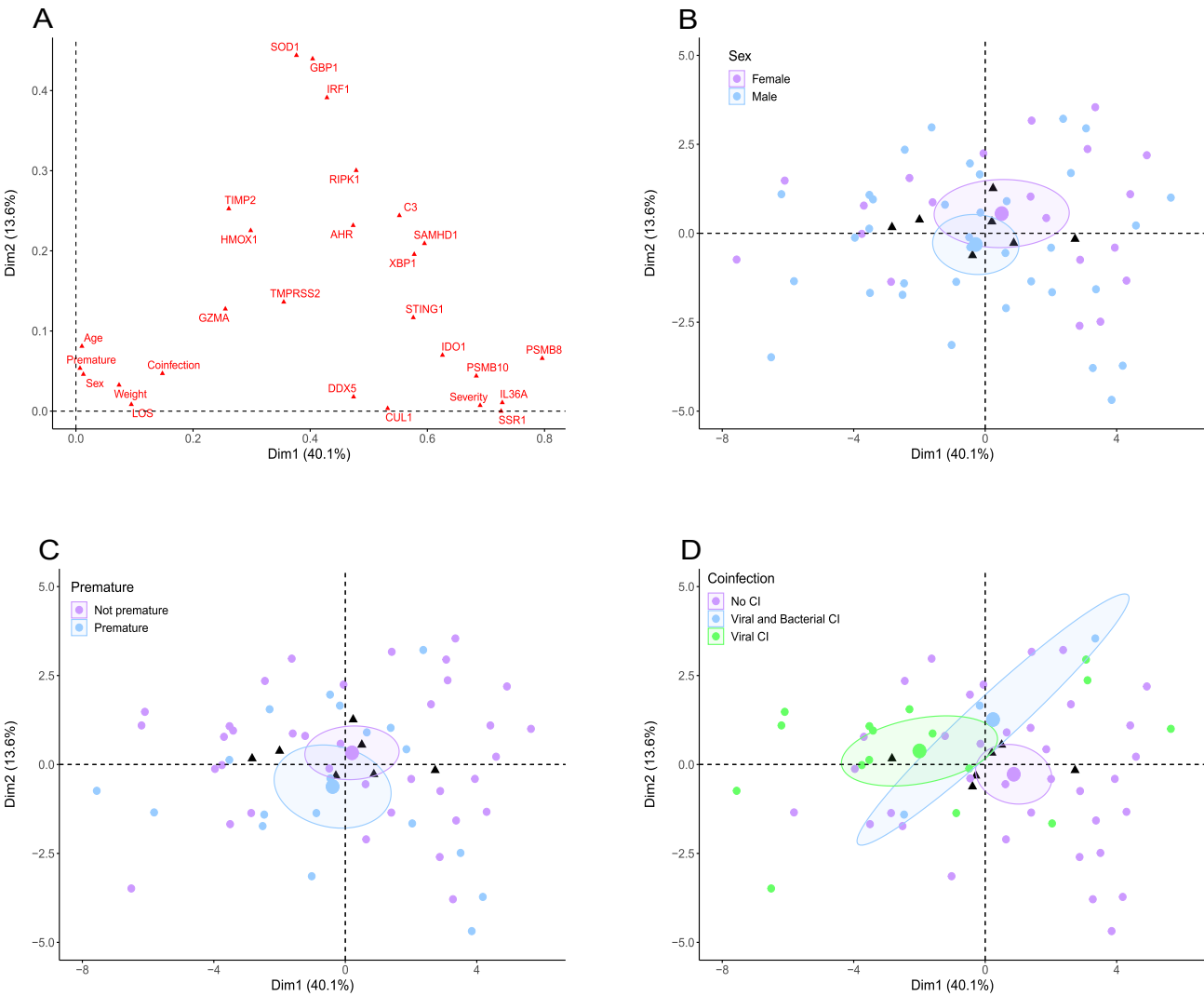

Supplement: Supplementary Data 2 [file mmc2.pdf]

Figure S3

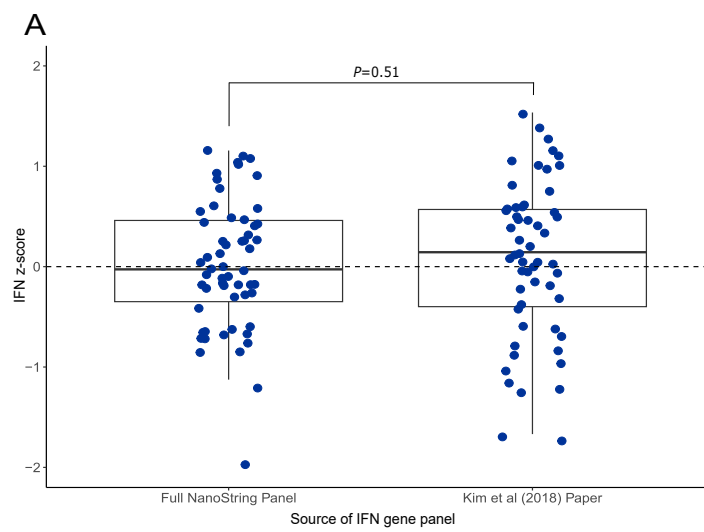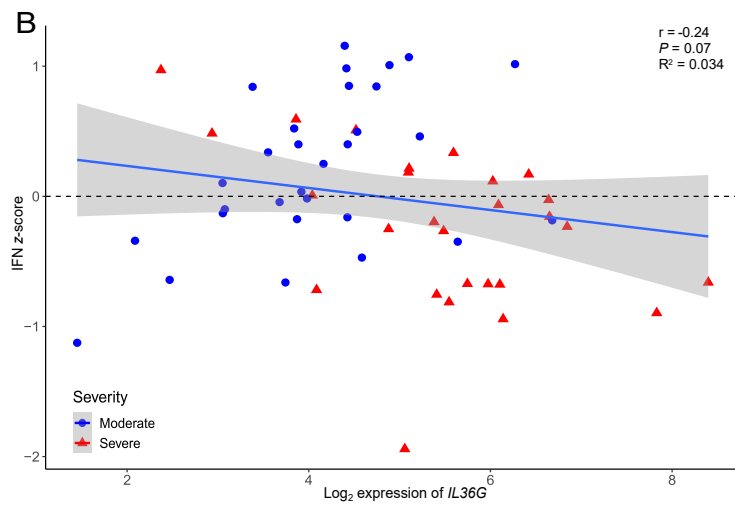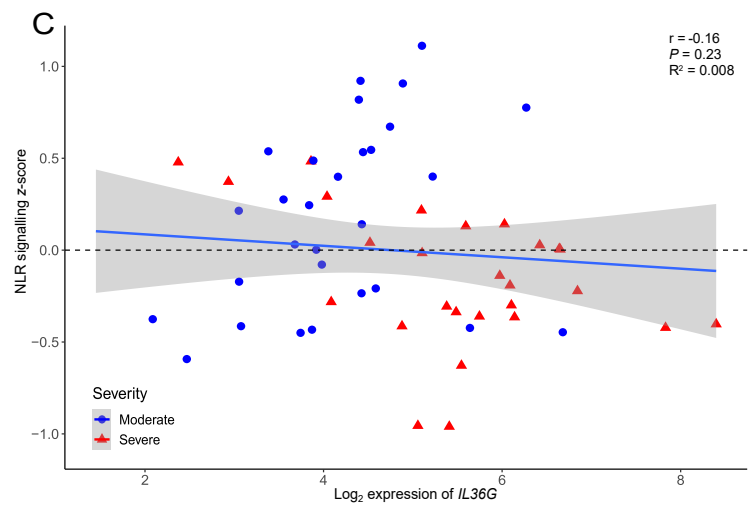

Supplement: Supplementary Data 3 [file mmc3.pdf]

Figure S4

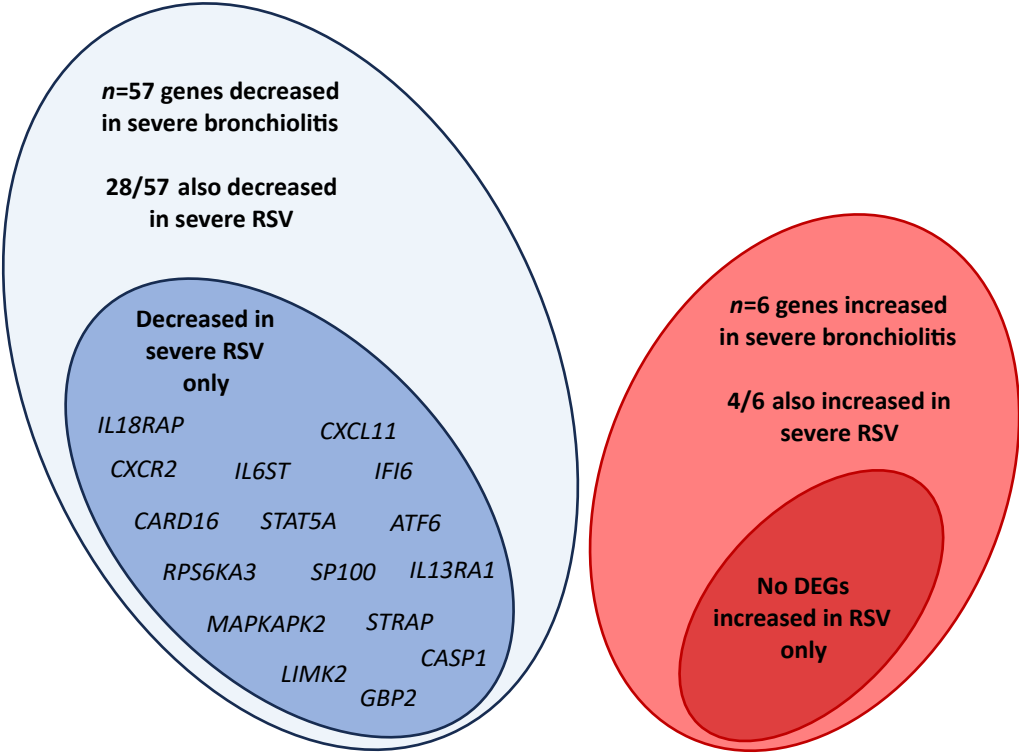

Supplement: Supplementary Data 4 [file mmc4.pdf]

Figure S5

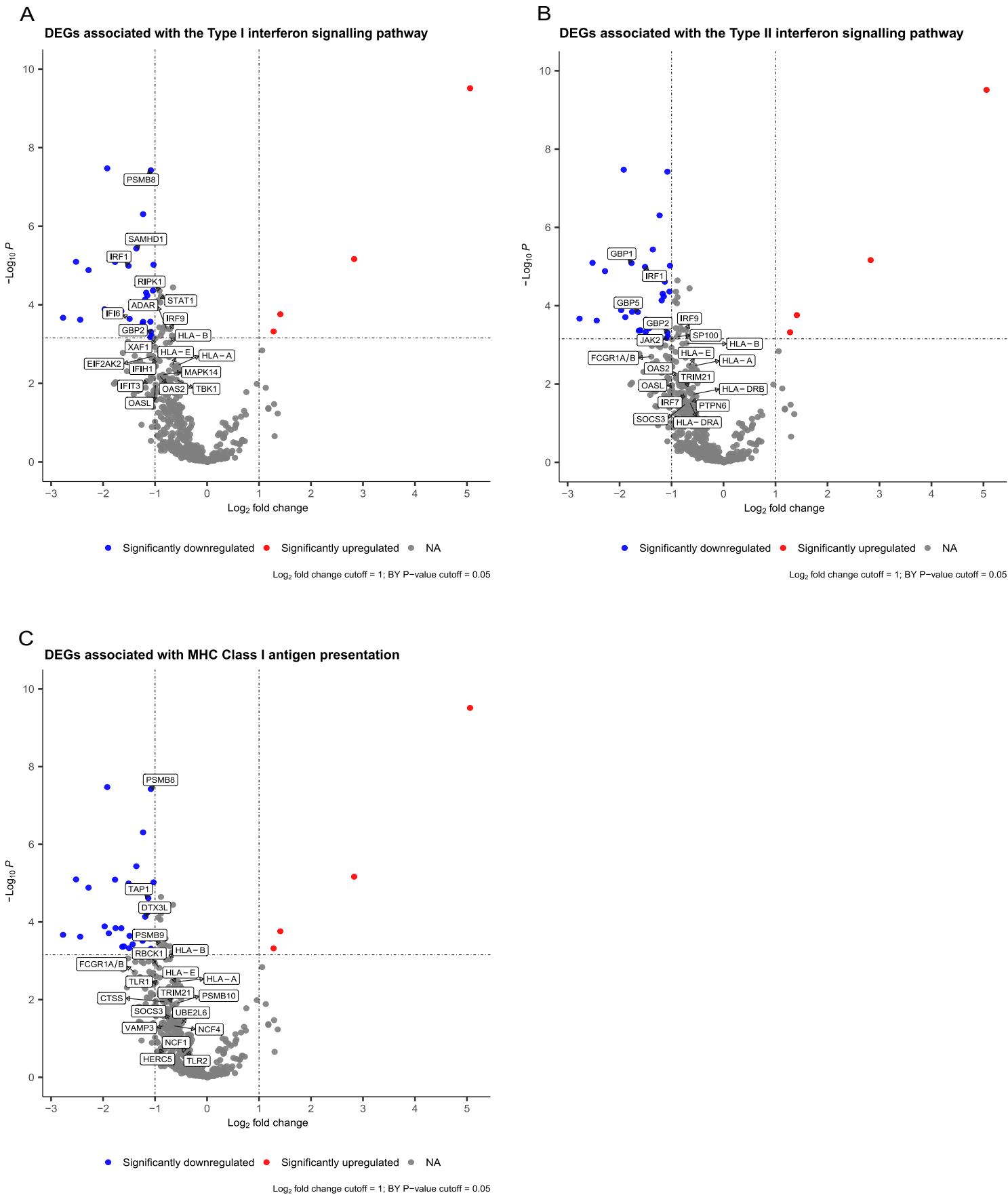

Supplement: Supplementary Data 5 [file mmc5.pdf]

Figure S6

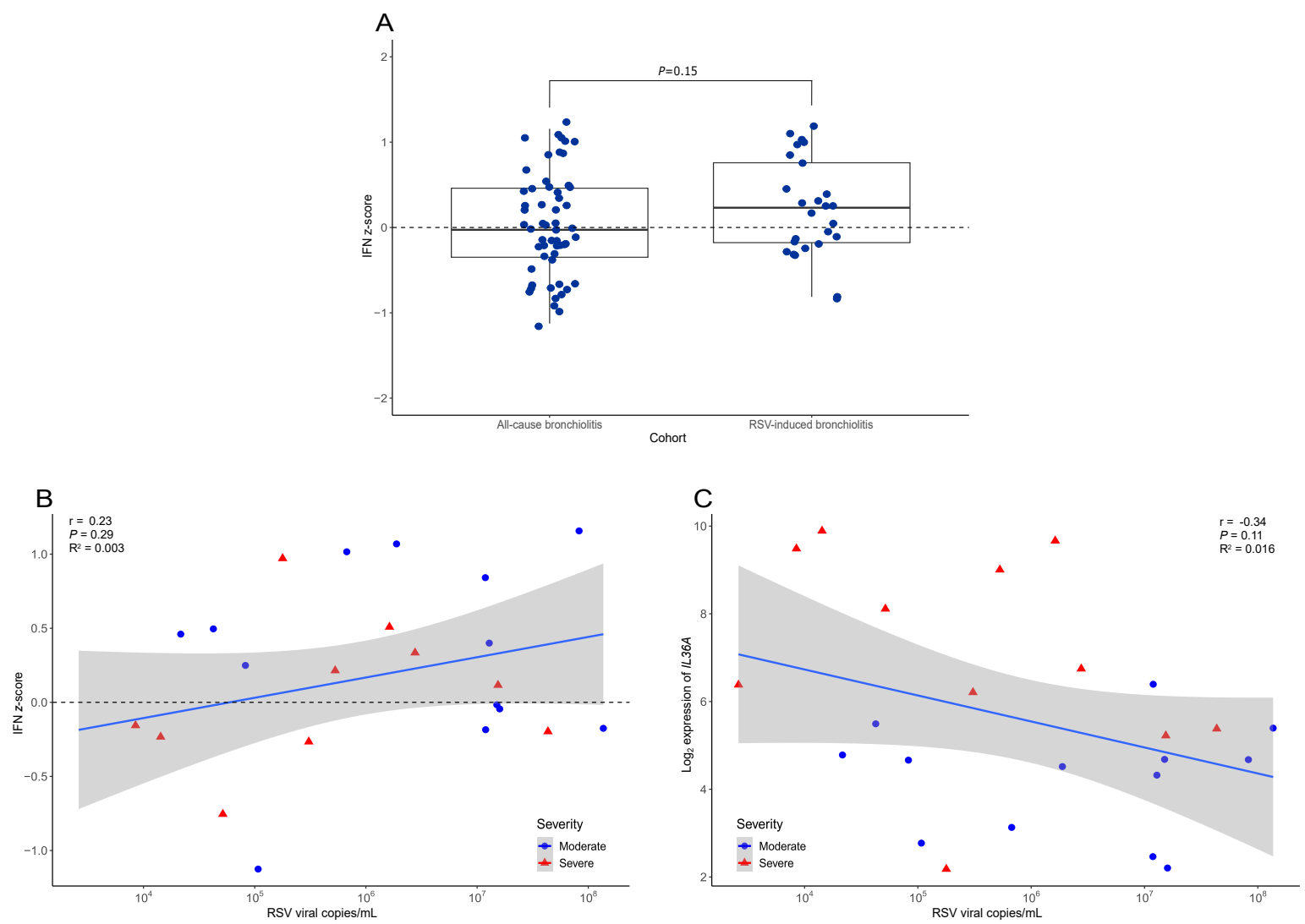

Supplement: Supplementary Data 6 [file mmc6.pdf]

Figure S7

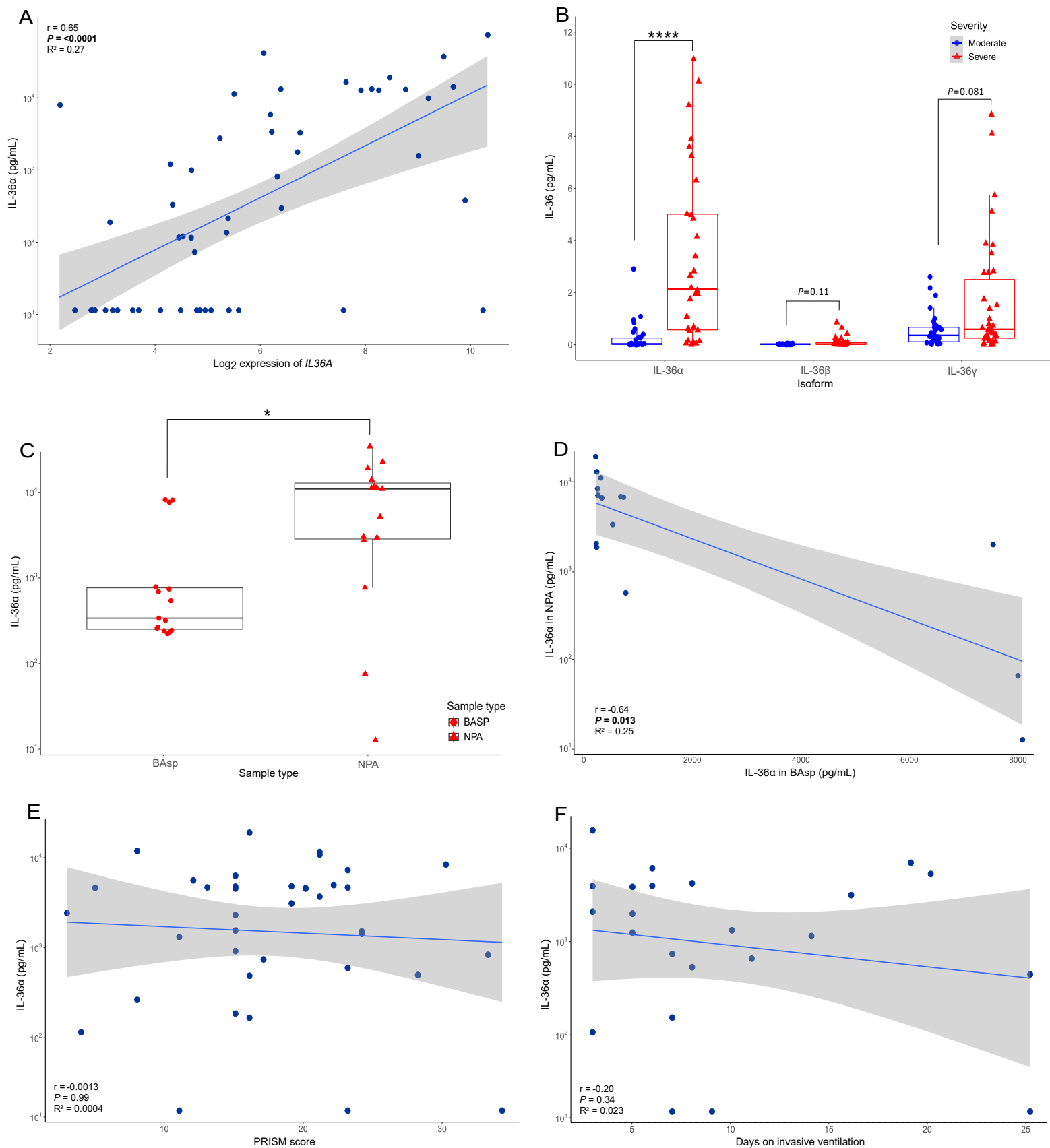

Supplement: Supplementary Data 7 [file mmc7.pdf]

Figure S8

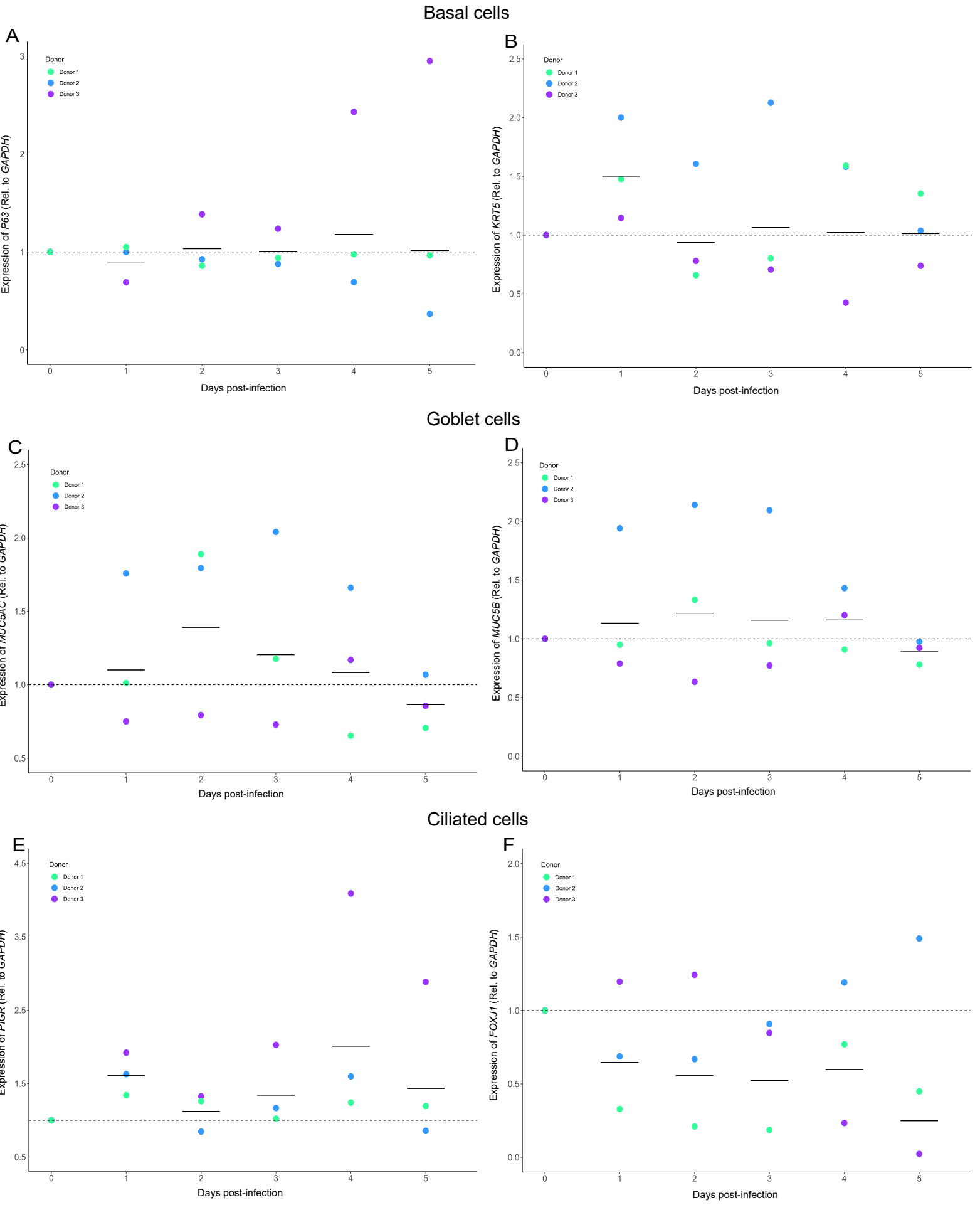

Supplement: Supplementary Data 8 [file mmc8.pdf]

Figure S9

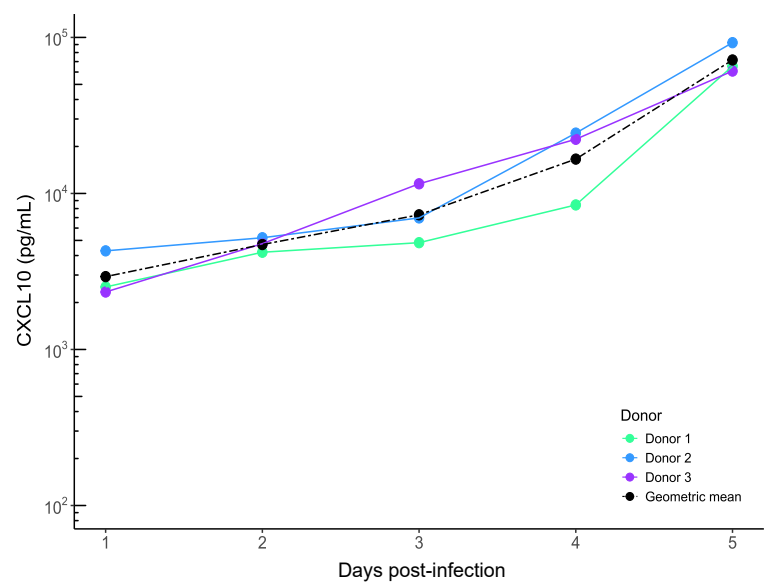

Supplement: Supplementary Data 9 [file mmc9.pdf]

Figure S10

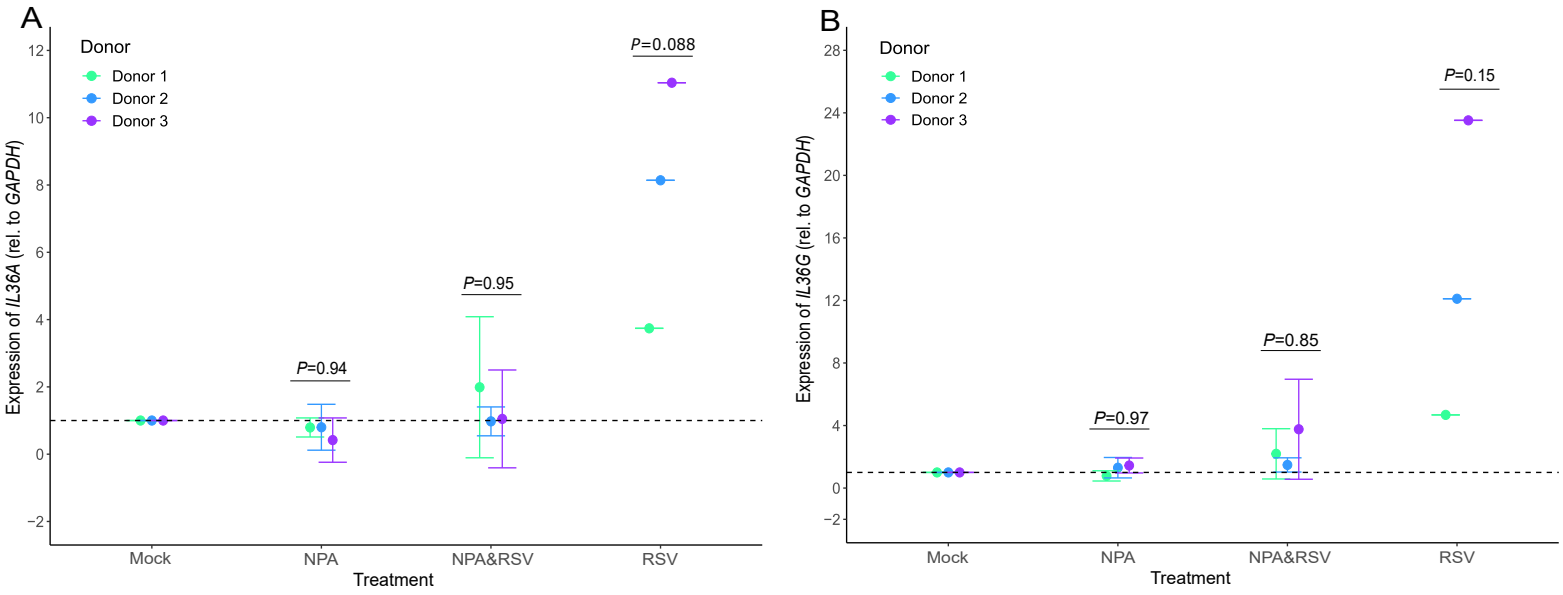

Supplement: Supplementary Data 10 [file mmc10.pdf]
