# Supplementary material for: Mucosal IL-36 is a defining feature of severe paediatric bronchiolitis
Source: Mucosal Immunol. 2026 Apr;19(2):1907–21. doi: 10.1016/j.mucimm.2026.01.012 (PMC13195398; doi:10.1016/j.mucimm.2026.01.012)
Supplement: Supplementary Data 11 [file mmc11.pdf]

Table S1: RSVSAM patients included in NanoString analyses (all-cause bronchiolitis):

	**Moderate** *n* **= 30**	**Severe** *n* **= 27**	***P*** **Value**
Age (days), median (range)	200 (23-630)	79 (14-630)	0.13
Sex, male:female	17:13	18:9	0.44†
**Weight (kg), median (range)**	**8.0 (3.56-15)**	**4.5 (2.3-13.7)**	**0.015***
**LOS (days), median (range)**	**2 (0.5-5)**	**8.0 (3-125)**	**<0.0001***
Time between symptom onset and sample collection (days), median (range)	4 (0-15)	4 (1-17)	0.37*
**Viral coinfections,** ***n*** **(%)**	**6/30 (20%)**	**13/27 (48%)**	**0.024†**
Bacterial coinfections, *n* (%)	4/30 (13%)	4/27 (15%)	0.87†
Prematurity, *n* (%)	9/30 (30%)	10/27 (37%)	0.57†
Comorbidity, *n* (%)	6/30 (20%)	6/27 (19%)	0.84†
PaO_2/FiO_2 score, median (range)	N/A	164 (60-282)	N/A
PRISM score, median (range)	N/A	16 (3-34)	N/A
Days on IMV, median (range)	N/A	7 (3-25)	N/A

Table S2: RSVSAM patients included in NanoString analyses (RSV(+) only):

	**Moderate** *n* **= 14**	**Severe** *n* **= 13**	***P*** **Value**
Age (days), median (range)	105 (23-480)	28 (14-330)	**0.024***
Sex, male:female	9:5	9:4	>0.99†
**Weight (kg), median (range)**	**6.0 (3.56-15)**	**3.9 (2.3-10)**	**0.015***
**LOS (days), median (range)**	**3 (0.5-5)**	**8.5 (4-74)**	**<0.0001***
Time between symptom onset and sample collection (days), median (range)	5 (1-9)	4 (1-8)	0.24*
Viral coinfections, *n* (%)	4/14 (29%)	6/13 (46%)	044†
Bacterial coinfections, *n* (%)	1/14 (7%)	0/13 (0%)	>0.99†
Prematurity	4/14 (29%)	2/13 (15%)	0.64†
Comorbidity, *n* (%)	1/14 (7%)	2/13 (15%)	0.60†
PaO_2/FiO_2 score, median (range)	N/A	161 (49-245)	N/A
PRISM score, median (range)	N/A	20 (8-33)	N/A
Days on IMV, median (range)	N/A	8 (8-14)	N/A
