# Supplementary material for: Mucosal IL-36 is a defining feature of severe paediatric bronchiolitis
Source: Mucosal Immunol. 2026 Apr;19(2):1907–21. doi: 10.1016/j.mucimm.2026.01.012 (PMC13195398; doi:10.1016/j.mucimm.2026.01.012)
Supplement: Supplementary Data 12 [file mmc12.pdf]

Table S3: Demographics of patients recruited to the ELLI study:

	**Healthy controls** *n* **= 11**	**Severe** *n* **= 29**	***P* Value**
Age (days), median (range)	146 (36-445)	67 (18-504)	0.089*
**Sex, male:female**	**11:0**	**17:12**	**0.008†**
**Weight (kg), median (range)**	**9.6 (2.8-12.4)**	**4.2 (3.2-10.6)**	**0.018***
**LOS (days), median (range)**	**1 (1-2)**	**6 (3-33)**	**<0.0001***
RSV status, RSV:Non-RSV	N/A	14:15	N/A
Viral coinfections, *n* (%)	N/A	9/29 (31%)	N/A
Bacterial coinfections, *n* (%)	N/A	4/29 (14%)	N/A
Prematurity, *n* (%)	5/8 (45%)	12/18 (66%)	0.44†
Comorbidity, *n* (%)	6/11 (54%)	10/29 (35%)	0.30†
Max FiO_2, median (range)	N/A	100 (40-100)	N/A
PIP score, median (range)	N/A	27 (16-33)	N/A
**Days on IMV, median (range)**	**1 (1-1)**	**6 (3-22)**	**<0.0001***
